# Supplementary material for: Estimating the transfer rates of bacterial plasmids with an adapted Luria–Delbrück fluctuation analysis
Source: PLoS Biol. 2022 Jul 25;20(7):e3001732. doi: 10.1371/journal.pbio.3001732 (PMC9352209; doi:10.1371/journal.pbio.3001732)
Supplement: Attachment — Submitted filename: Comments_PBIOLOGY-D-22-00314.pdf [file pbio.3001732.s002.pdf]

Comments on PBIOLOGY-D-22-00314 (Kosterlitz et al.)

By providing an improved method of estimating conjugation rates (as well as carefully comparing it to previous methods), this study fills an important gap in the field. It is also explained clearly and with enough detail, such that others could apply the method themselves. Kudos to the authors for a very interesting, thorough and rigorous study. They've done a great job of keeping the main text concise and focused on the key messages, but the Supplementary contains a wealth of useful additional information – I appreciate the amount of work that goes into this material, and I think it really contributes to good science. The manuscript is already very clearly written and the figures are well designed to get across some rather complex ideas – clearly a lot of thought has gone into presentation.

Scientifically, I don't see any faults with this study – the math all looks correct, and the experimental protocol is thoroughly described and includes carefully chosen controls.

On close reading of both main + SI, I've still ended up with a long list of minor suggestions to further improve the presentation or clarify occasional details, but I emphasise that this is pretty high-level polishing and I am happy to leave most of these suggestions to the authors' discretion (other than fixing an apparent broken link in the GitHub repository).

1. Abstract – I personally thought the importance of using a stochastic approach could have been better emphasised – the current framing suggests the main advance is to address asymmetries in rates, with stochasticity an afterthought rather than a crucial factor in achieving accuracy. I had a similar impression in the Introduction – it wasn't clearly stated why a stochastic description is important in general, not only for unequal rates. For example, what if $\gamma_T = \gamma_D$ but both are low? (However, if the authors consider the emphasis on heterogeneous rates to be more relevant to the field, then I accept their judgement on this point.)
2. L45-6: the wording "not being affected by different growth and transfer rates" could be confusing; I think you mean the *method* is equally applicable, not that the *results* are unaffected.
3. Eq. [4]: should the LHS just be γ, not γ_D?
4. L226, wording could be slightly clearer: "where there are *initially* no transconjugants"; "the only process that can *initially* change the number of transconjugants" (or, "produce the first transconjugant")
5. A critical assumption in the derivation of the p_0 estimation method (similarly to its use for mutation rate estimation) is that the mutant or transconjugant population, once becoming non-zero, never drops to zero again. In the present case, there are (at least) two key assumptions for this to be true: (1) no cell death; (2) no segregative loss of the plasmid. #1 should be reasonable in benign growth conditions but could be given a passing mention. #2 seems important, and was actually addressed in the Supplementary – I think this is well worth mentioning in the main text.
6. L248-9: Using the Gillespie algorithm, presumably you have treated all populations (D, R, T) stochastically? This would be helpful to state explicitly, as it contrasts with the analytical treatment above, where only T is stochastic.
7. L266-7: The LDM estimate really requires a mix of presence/absence across replicates.
8. L289 word choice: "surpassed" might be confusing (suggesting the estimate was higher); rather, "outperformed"?
9. L304: "at a time close to *the average* t*"

10. Around L339: Is there any risk that wells were already grown enough to be (somewhat) turbid before addition of the selective media? If so, will turbidity decline as cells die, or could dead cells or debris continue to contribute enough OD to interfere with scoring turbidity at the end of the assay?

11. As I was reading the main text, a couple of questions about the lab protocol came to mind: (1) Are both estimation methods (SIM and LDM) applied to the exact same cultures, or separate ones? (2) Were the growth rates (ψ's) estimated separately? These points became clear later in the more detailed methods, but might be worth a brief mention when they first come up. Also, while I appreciate that \tilde{t} is chosen differently for each parameter set, I would find it helpful to have some idea of the range in the main text (at least in Materials & Methods, around L604) and perhaps in relevant figure captions.

12. L417: "the Levin *et al.* model" needs a reference number. This is also the first time this model is mentioned, and its key feature(s) or relation to the SIM could be briefly explained.

13. Around L460: SI section 8 analytically considers variance in the LDM to the ASM, whereas Fig. 4 compares LDM to SIM. Do the analytical insights transfer? Is SIM a special case of ASM if there is no resource depletion?

14. L614 minor semantic point: The medium inhibits donors & recipients (absolute growth rate < 0) while *allowing growth* of transconjugants (absolute growth rate > 0). Selection arises as a result of this combination – but selection (due to a *relative* fitness advantage) is not synonymous with growth (in absolute numbers); it's also possible to have a selective advantage while declining in absolute terms. (Similar issue in SI, L684: selection for resistance should not be equated with concentrations below the resistant strain's MIC.)

15. L623: Are colony counts from each of these three wells pooled or averaged to get a single estimate of population density?

16. L658: Clarify (also in the Fig. 4 caption) that the SIM method only shows 100 estimates, not 10'000.

17. L671: "a single incubation time" in what sense? It sounds like it differs for each parameter setting, and between LDM and SIM methods.

18. GitHub repository: This is a great addition, but some of the links seem to be broken. If I click on Supporting Data or Simulations, I get only a garbled Readme.md file (see screenshot below). Please check/fix this before publication. (I'm on a Safari browser, if that makes any difference.)

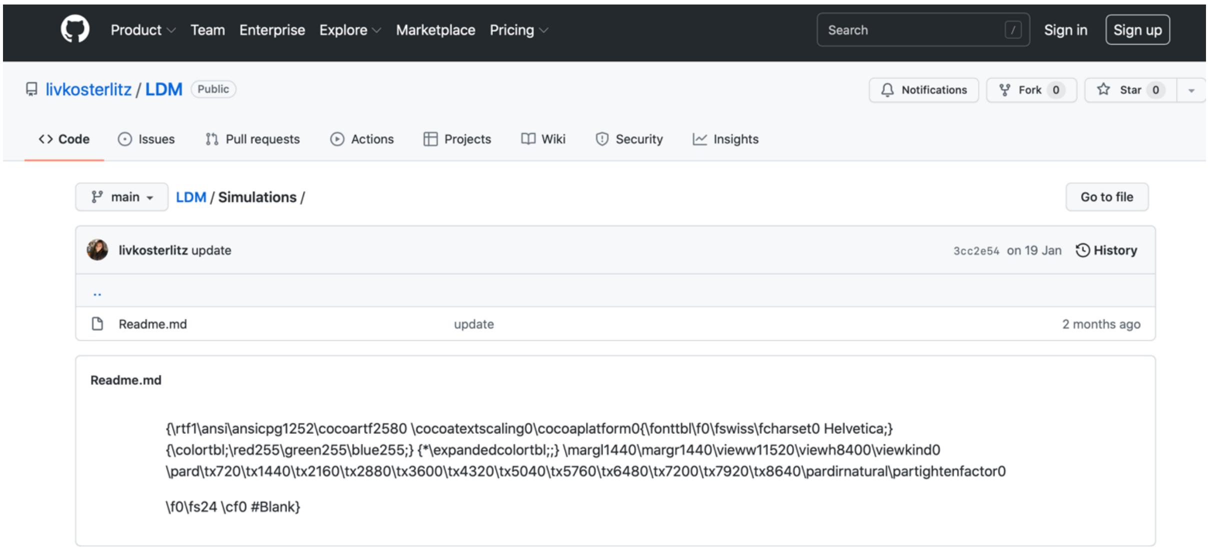

19. Fig. 3 caption, around L750 (and similarly for Fig. 4 caption): State how many estimates are derived from these 10'000 simulations.
20. Fig. 4 caption, L765: incubation times are specific to each parameter setting – and also differ between LDM and SIM?
21. To play devil's advocate, the superior precision of the LDM method might be expected given that the estimate is derived from a much larger sample size of populations. For instance in Fig. 4, each LDM estimate is based on 100 simulated populations while each SIM estimate is based on only one. Similarly, in SI section 8, the variance in the ASM estimate is derived for a single measurement of T_t (I think) whereas the variance in the LDM estimate is derived for W wells (plotted in SI Fig. 10 for W=10 and W=100). Multiple replicates are clearly necessary for an estimate based on a stochastic process, but the authors might acknowledge that the improvement in precision comes at the cost of doing a larger experiment (or rebut my thinking if you disagree). To that end – the authors could also consider pointing out, when describing the experimental protocol, that the number of replicate wells used to derive one estimate in the LDM can be varied; 84 is convenient for the layout of a single microtitre plate, but there is no reason a user couldn't adjust this, either decrease (at the cost of lower precision) to run higher-throughput assays across multiple environments, or increase to improve precision.
22. SI section 1a, L52-7: I'd find it helpful to state what these assumptions mean in terms of model parameters (i.e. ψ=0 and D, R constant?).
23. SI table 4, 5th row: "per replicate" is a confusing word choice here – perhaps, "to obtain one estimate"?
24. SI Fig. 5 – clarify which model has been simulated (equations 4.1-4.4?). Also add $\tau_D = \tau_T = 0$ (I assume) to the list of baseline parameter values.
25. SI sections 4f-4h: I'd find it helpful to introduce these sections by briefly explaining the motivation/aim(s) of the following analysis. Similarly, at the end of section 4h, it would be helpful to add a conclusion – is there some additional insight into interpretation of experimental data?
26. SI Fig. 6 caption (L512, 514): "significantly" has connotations of having done a statistical test – rather say "substantially"?
27. SI section 6b – around L636/SI Fig. 8b: the growth rate varies over time – which value was taken as the final estimate? The maximum?
28. SI section 6c – please clarify, does the ratio of [Strep]/[Tet] remain constant in dual-antibiotic medium? (I.e. it's not a two-way gradient?)
29. SI section 6d, L723: just to check, was the final density = 4 x 10^-7 x initial density (i.e. dilution factor 0.25 x 10^7) or was the dilution factor 4 x 10^7?
30. SI section 6d, around L761: I'm confused about why this issue arises – why is the recipient colony count so low on plates supplemented with antibiotic selecting for the recipient?
31. L807: "10^4, 10^5, and 10^6-fold *dilution*" (missing word)
32. SI section 7 – Again, I'd find it helpful to start off with a statement of motivation/aim for this section.
