# Supplementary material for: Estimating the transfer rates of bacterial plasmids with an adapted Luria–Delbrück fluctuation analysis
Source: PLoS Biol. 2022 Jul 25;20(7):e3001732. doi: 10.1371/journal.pbio.3001732 (PMC9352209; doi:10.1371/journal.pbio.3001732)
Supplement: Attachment — Submitted filename: LDM_response_letter.pdf [file pbio.3001732.s003.pdf]

May 6, 2022

Senior Editor, *PLOS Biology*

Dear Dr. Roberts,

Thank you for the opportunity to revise our manuscript entitled “Estimating the rate of plasmid transfer with an adapted Luria–Delbrück fluctuation analysis,” for submission as a Methods and Resources article to *PLOS Biology*. The comments provided by the reviewers were very thoughtful and have led to an improved manuscript.

Below we offer detailed point-by-point responses to each of the reviewers’ suggestions. We include each original suggestion in black text, and our response in blue text. Any changes we made within our manuscript to address reviewer comments are highlighted in yellow. In some cases, a given change (including addition of new sections) was made in response to the suggestions of multiple reviewers. In those cases, the same text, figure, or section is presented under each relevant comment in different reviewers’ sections below. We realize this causes our response to be long, but we hope this makes things easiest for each reviewer as they work through our responses.

Thank you to you and the reviewers for your help in making this a stronger manuscript. If you have any other questions or concerns, please do not hesitate to contact us.

Sincerely,

Olivia Kosterlitz
Adamaris Muñiz Tirado
Claire Wate
Clint Elg
Ivana Bozic
Eva M. Top
Benjamin Kerr

Reviewer 1:

The authors have developed a new method to estimate plasmid conjugation rates that explicitly accounts for the stochasticity inherent in conjugation. This is a fundamentally new method, and a refreshing take on estimating conjugation rates. I think this will be a useful tool for microbiologists and a good starting point for further work taking into account the stochasticity of conjugation.

I would also like to congratulate the authors on their development of the paper since the very first preprint version.

We sincerely thank the reviewer for this positive assessment.

However, I see several points that could further improve this paper and heighten its impact.

-- General comments: --

1. The LDM method is unique in its focus on the stochastic, early dynamics of conjugation. I am missing some more discussion of the biases unique to these early measurements. The authors already discuss the transconjugant extinction probability (in the presence of antibiotics), but it is a bit hidden in the supplementary materials. I did not see any discussion of dilution or plating errors (which may be greater at smaller population sizes), nor population level heterogeneity in resumption of growth when starting the conjugation assay. How do these stochastic dynamics affect the LDM in a way that is similar/different to other methods?

We thank the reviewer for raising these issues. We thought these points were important enough to explore through further simulations, and we have added a new SI section that is explicitly focused on the added random effects of dilution, plating, and probabilistic failure of cells to form colonies or establish lineages in liquid culture. The bottom line from this section is that these random effects do increase variance of estimates and appear to impact the SIM estimate to a greater extent than the LDM estimate. We include this new SI section below for the reviewer's convenience:

SI Section 9: Random effects on estimate accuracy and precision

In this section we explore, through simulation, some of the consequences of other random effects on the LDM and SIM estimates. Some of these effects are a consequence of experimental protocols. For instance, both approaches require dilution and plating in the laboratory to estimate donor and recipient density (and the SIM approach also uses dilution and plating to estimate transconjugant density). Because dilution and plating are subject to random sampling effects, there will be density-estimation errors introduced by these procedures. Other random effects are features of the cells under study. As we describe in SI sections 6d and 7, there can be a non-zero probability that any cell will fail to establish a lineage. For instance, a donor cell may fail to form a colony on a plate after incubation on selective medium, or a lone transconjugant cell in a well may fail to yield a turbid culture after incubation in selective medium. Again, there will be stochasticity in the number of cell lineages that go extinct, which will lead to error in calculating key quantities needed for the estimates (even with corrections). Here we explore the consequences of some of these random effects.

Random effects in dilution, plating, and failure to form colonies: We ran our stochastic simulations as before (SI Section 4), but instead of using the simulated numbers of cells directly for our estimates, we wrote a dilution-plating subroutine to simulate how cell density would be gauged in the lab. Suppose that a cell population has an actual density of N_0 cells/mL. A 10-fold dilution series is generated recursively by diluting 100μL into 900μL. Thus, the density of cells in the first dilution is:

$$N_{-1} = rv[\text{Poisson}(0.1N_0)]$$

where $rv[d]$ is a random value for a variable with a distribution given by d. The density of cells in the second dilution is:

$$N_{-2} = rv[\text{Poisson}(0.1N_{-1})].$$

More generally, the ith dilution has density:

$$N_{-i} = rv\big[\text{Poisson}\big(0.1N_{-(i-1)}\big)\big]$$

Now 100μL of each dilution in the entire series is plated, where the number of bacterial cells from the ith dilution landing on the plate is:

$$B_{-i} = rv[\text{Poisson}(0.1N_{-i})]$$

Finally, the number of colonies forming (given an extinction probability of π) on the ith dilution plate is:

$$C_{-i} = rv[\text{Binomial}(B_{-i}, 1-\pi)]$$

We pick the dilution plate with the maximum number of colonies in the range between 30 and 300. If every dilution plate is below 30 colonies, we simply use the plate with the maximum number of colonies. For generality, let's suppose we select the ith dilution plate. We compute the cell density of the undiluted culture as:

$$N_{\text{est}} = \frac{C_{-i}}{1-\pi} \times 10^{i+1} \ \frac{\text{cells}}{\text{mL}}$$

Given the random effects of dilution, plating, and cell lineage extinction, it is likely that N_{est} will deviate from the actual cell density N_0.

For the SIM estimate, we use this procedure to generate the density of donors, recipients and transconjugants that are used in the estimate. For the LDM estimate, we use this procedure to generate the density of donors and recipients that are used in the estimate. Also, if the extinction probability of transconjugants in the wells is non-zero, we must also track a monoculture of transconjugants in order to estimate the transconjugant growth rate needed for the LDM correction (equation [7.1]), and we use the above procedure to estimate the transconjugant densities in these cases.

Random effects in wells with transconjugants: However, we also need to calculate the fraction of wells with transconjugant-selecting medium that are not turbid for the LDM estimate. Here the actual simulated number of transconjugants in a given population at the end of the assay is $T_{\tilde{t}}$. The number of lineages that do not go extinct is

$$L_{-i} = rv[\text{Binomial}(T_{\tilde{t}}, 1-\pi)]$$

If $L_{-i} > 0$, then the well is turbid, whereas if $L_{-i} = 0$, then the well is non-turbid. The proportion of non-turbid wells out of a total of W wells (P_{nt}) can then be calculated. If we have this quantity and all the relevant cell densities, we can then use equation [7.2] to calculate the corrected LDM estimate.

Results: We show the results of adding these random effects in SI Figure 12. Each rectangle represents 100 estimates for a combination of the time of the assay ($\tilde{t}$) and an extinction probability (π), which, for simplicity, we assume is the same for all cell types both on plates and in wells. For reference, estimates without the random effects of dilution, plating, and extinction are given in the bottom row of each plot. Estimates with the random effects of only dilution and plating can be found in the row with zero extinction probability in each plot. We note that as the extinction probability increases, the end point of the assay must also increase (to obtain sufficient colonies and turbid wells), thus, the range of times shift with this quantity.

As random effects are added, both the LDM and SIM estimates of the donor conjugation rate tend to deviate more from the actual value, but there is not systematic deviation (SI Fig. 12a). Not surpisingly, as random effects are added, the variance in estimates rises, but this effect is more pronounced for the SIM estimate (SI Fig. 12b). For both approaches, a zero estimate is possible (when there are no transconjugant colonies or no turbid transconjugant wells) and for the LDM estimate an infinite estimate is possible (when all the transconjugant wells are turbid). However, we see these extreme values occur primarily at the boundaries of the time interval for the end of the assay (SI Figs. 12c and 12d).

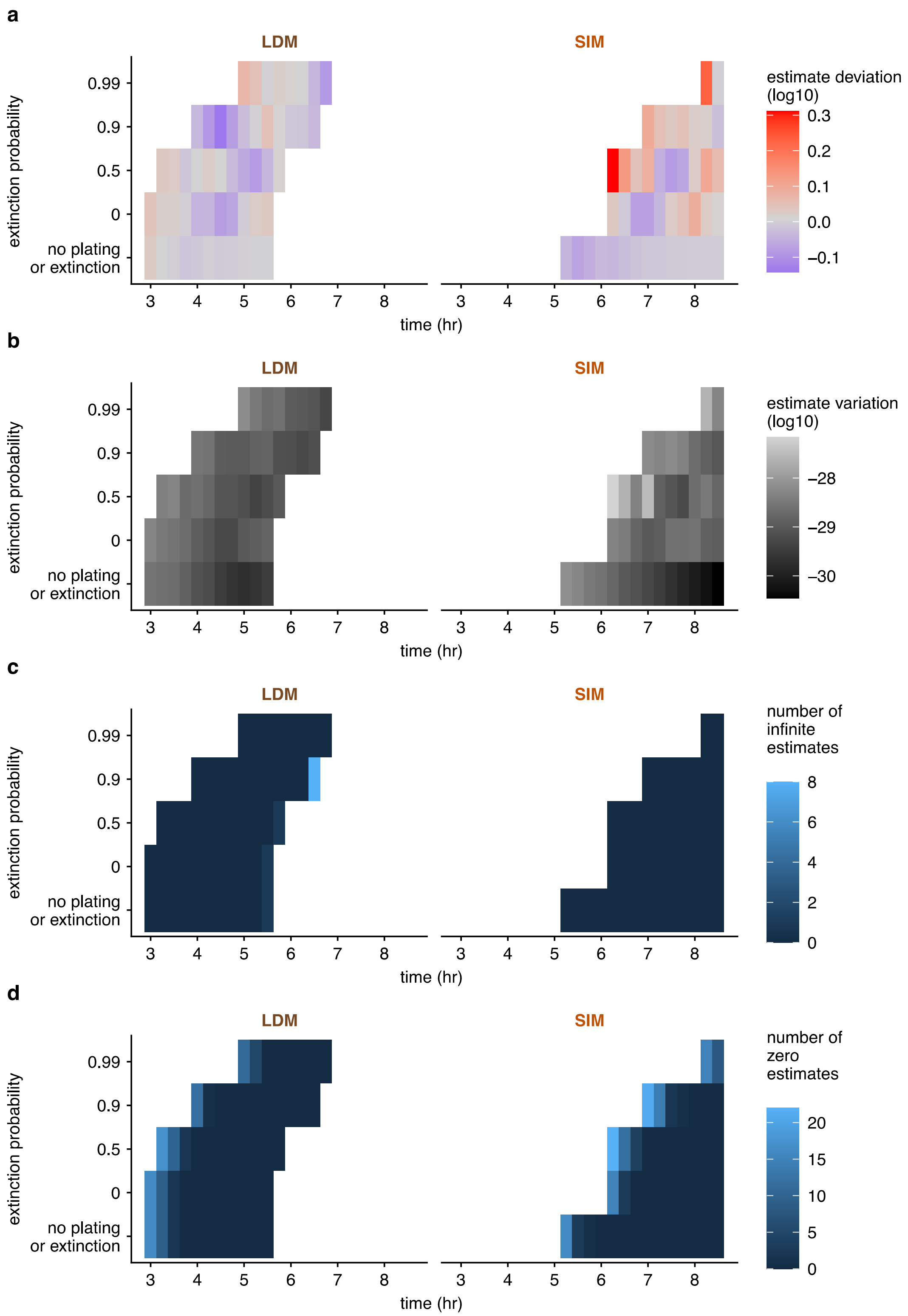

SI Figure 12: The random effects of dilution, plating, and failure to establish on the accuracy and variance of the LDM and SIM estimates. Different extinction probabilities are used, as indicated. The parameter values and initial densities are the same as SI Figure 5a which were $\psi_D = \psi_R = \psi_T = 1$ and $\gamma_D = \gamma_T = 1 \times 10^{-14}$. The dynamic variables were initialized with $D_0 = R_0 = 10^5$ and $T_0 = 0$. The

scenario with no dilution plating and a zero-extinction probability (the bottom row in each panel) is the data from SI Figure 5a. The mean deviation (a) and variation (b) of each set of estimates is given at 15-minute time intervals where at least 75 out of the 100 calculate estimates produced a finite non-zero value. (c) The number of infinite estimates out of the 100 calculated in the relevant intervals. (d) The number of estimates with a zero value out of the 100 calculated in the relevant intervals. We note that the Gillespie algorithm is computationally expensive when the densities get very large. Therefore, due to the longer incubation times needed for the SIM, only 100 populations of the 10,000 were simulated through the later time intervals until on average a population density of 1 x 10^9 is reached (i.e., $\tilde{t}$ = 8.5 h). The remaining 9,900 populations, used to compute $\hat{p}_0(\tilde{t})$ for the LDM, were run until an average of 100 transconjugants was reached (i.e., $\tilde{t}$ = 6.9 h). This explains the truncation of the SIM estimates at 8.5 hours and the LDM estimates 6.75 hours, which is most notable in the scenario where the extinction probability is 0.99.

The reviewer also asks about the effects of population-level heterogeneity. While we don't include these preliminary results in our new SI section, we thought we would share them here (we hope to return to these effects in future work). In a new set of runs, for every population simulated, we picked the logarithm of growth and transfer parameters from a normal distribution centered on the logarithm of specific "baseline" parameter values. Thus, every simulated population could differ in these parameters. We simulated two levels of heterogeneity, and the results are in the figure below. Population-level heterogeneity does lead to slight bias and increased variance in the estimates. We note that these effects are more pronounced for the SIM estimate than the LDM estimate.

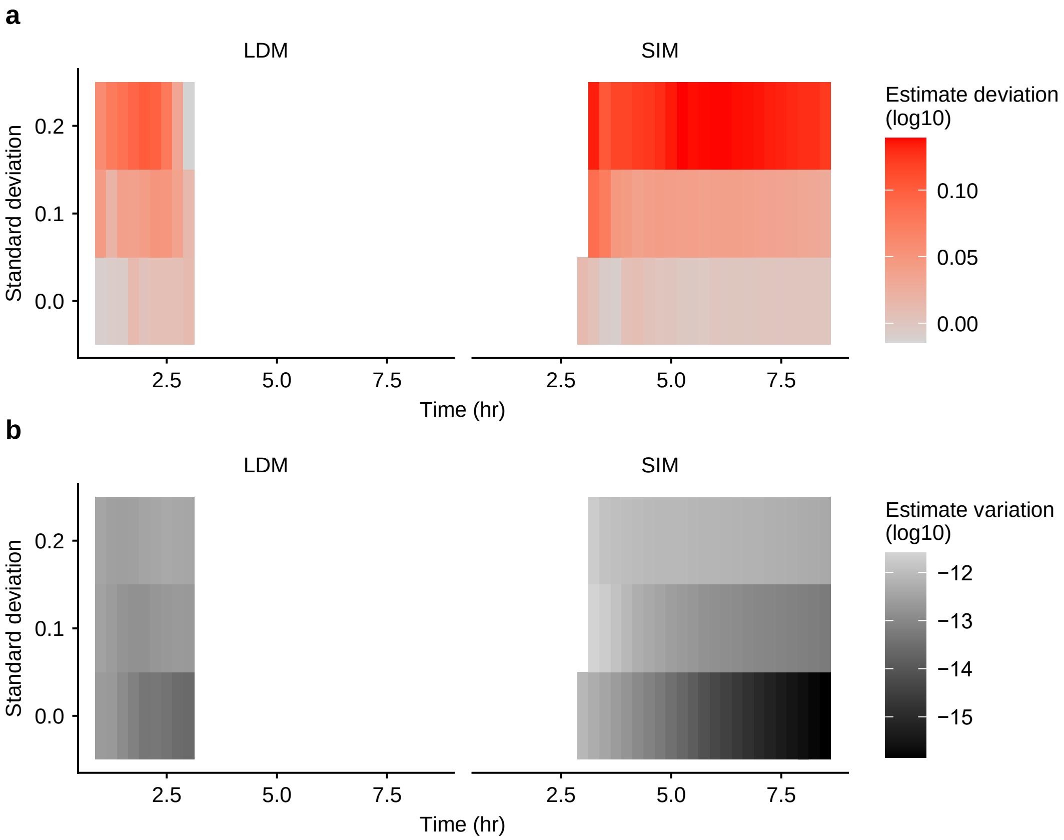

Figure: The effects of heterogeneous parameters on the accuracy and variance of the LDM and SIM estimates. The baseline parameters used here are the same as in SI Figure 1 which were $\psi_D = \psi_R = \psi_T = 1$, and $\gamma_D = \gamma_T = 10^{-6}$. The dynamic variables were initialized with $D_0 = R_0 = 10^2$ and $T_0 = 0$. The scenario with no variation added (the bottom row in each panel) is the data from SI Figure 1. For each

variable growth rate $\psi_X{}'$ (where the prime indicates a specific co-culture population) we pick the value such that $\ln \psi_X{}' \sim Normal(\ln \psi_X, \sigma^2)$ where the unprimed parameter is the baseline value and $X \in \{D, R, T\}$. For each variable conjugation rate $\gamma_Y{}'$ we pick the value such that $\log_{10} \gamma_Y{}' \sim Normal(\log_{10} \gamma_Y, \sigma^2)$ and $Y \in \{D, T\}$. The parameter σ is the standard deviation of the normal distributions used, which gauges the degree of population-level heterogeneity. The mean deviation (a) and the variation (b) of each set of estimates is given at 15-minute intervals where at least 90 out of the 100 calculated estimates produced a finite non-zero value (the default filtering criteria for time sweeps described in the Materials and Methods).

2. In the stochastic simulations different numbers of simulations are compared for the different methods, since the LDM requires a panel of 84 simulations to be run for a single p0 estimate (line 652-658). This should be made equal to truly compare the variability of the different methods. From the description it was also not directly clear whether p0 was computed independently multiple times or reused across simulations to estimate the LDM. I would suggest comparing 1000 simulated conjugation assays of TDR / SIM / ASM against 1000 x 84 simulations to estimate the LDM.

We agree with the reviewer that we should clarify the comparisons. For simplicity, we changed all the relevant figures (Figure 4, SI Figure 1-4) to show 100 estimates of each approach. Given the computational burden of the Gillespie algorithm and the number of treatments in the manuscript, we didn't compare 1000 estimates of each as the reviewer suggested, as this would have required generating an extra 90,000 populations on top of the 10,000 already generated for each treatment. This is because we need 100 independent simulations to calculate $\hat{p}_0(\tilde{t})$ since each calculation of $\hat{p}_0(\tilde{t})$ is independent. In other words, the simulated populations are only used once (i.e., 100 LDM estimates requires 10,000 simulations to generate 100 independent $\hat{p}_0(\tilde{t})$ values).

To reflect this change and clarify our methodology, we have made the following alterations (highlighted words) to the relevant section (lines 699 – 705):

"The LDM estimate needs multiple populations to calculate $\hat{p}_0(\tilde{t})$; therefore, for each LDM estimate we reserved 100 independent populations to compute $\hat{p}_0(\tilde{t})$ then one random population in the set of 100 was used to calculate the initial and final cell densities. In other words, the 10,000 populations yielded 100 LDM estimates. In contrast, one simulated population yields one SIM estimate. Therefore, we used the random population chosen to calculate the densities for each of the 100 LDM estimates to calculate 100 SIM estimates."

We also made the following changes (highlighted words) to the relevant legends.

Lines 801 – 803 from Figure 4 legend:
"The Gillespie algorithm was used to simulate population dynamics. 100 estimates of the donor conjugation rate are shown for each parameter combination (summarized using boxplots with the same graphical convention as in Figure 3)."

SI lines 357 – 359 from SI Figure 1:
"The Gillespie algorithm was used to simulate population dynamics. 100 estimates of the donor conjugation rate are shown for each parameter combination (summarized using boxplots with the same graphical convention as in Figure 3)."

3. The substantial difference between the SIM and LDM on the experimental data (Fig. 6) is surprising, and I am missing the base case that shows they perform the same on a scenario where they should both do well (i.e. same growth/conjugation rates). The corresponding interpretation of the results (Line 367-382) also feels highly speculative. In the Figure 6 I further find it confusing that the cross-spp. SIM truncated is not listed next to the other cross-spp. results; and that there is no within-sp. SIM result.

I think this is an important point because the current Fig gives the feeling that "different methods simply give different results", which would hinder the broader adoption of the LDM and the comparison of results across the literature.

We agree with the reviewer that adding in an experiment where the SIM and LDM approaches would be predicted to both perform well should help with understanding (and hopefully adoption of the LDM approach). Thus, we added an experiment where we performed the truncated SIM protocol on the within-species mating. In alignment with our expectations under near parametric homogeneity (e.g., $\gamma_D \approx \gamma_T$), the SIM and LDM are congruent (i.e., we do not find statistically significant differences). In other words, when assumptions of both methods are met, the two approaches produce independent but similar experimental estimates of the donor conjugation rate. We added this experiment as the last boxplot in Figure 6. We note that we decided to retain the overall ordering of the boxplots in Figure 6 so that the order in which the comparisons are presented in the text is in alignment with the figure. To reflect this experimental addition, we have made the following highlighted word changes to the last paragraph in the relevant section (lines 430 – 434), the figure, corresponding figure legend (lines 839 – 849):

"Next, we performed the within-species mating between *E. coli* strains. The LDM estimate for within-species conjugation rate (within *E. coli*) was higher than the cross-species LDM estimate by almost six orders of magnitude (comparison B in Figure 6; t-test, p<0.001), a difference that could explain the inflated SIM estimate. To further explore this explanation, we performed an additional cross-species SIM experiment with a shorter incubation time. In Figure 3c, as the incubation time was shortened, the SIM estimate approached the LDM estimate of the donor conjugation rate. Running the SIM protocol with a truncated incubation period (5 hours) resulted in a significantly lower cross-species conjugation rate estimate relative to the standard SIM estimate (comparison C in Figure 6; t-test, p<0.05), a result consistent with the pattern predicted under heterogeneous conjugation rates. Under a scenario with reduced parametric heterogeneity (e.g., $\gamma_D \approx \gamma_T$), we predicted that the SIM and LDM estimate would be similar. Consistent with our prediction, the SIM estimate for the within-species conjugation rate using the truncated SIM protocol was not significantly different than the LDM estimate (comparison D in Figure 6; t-test, p=0.23)."

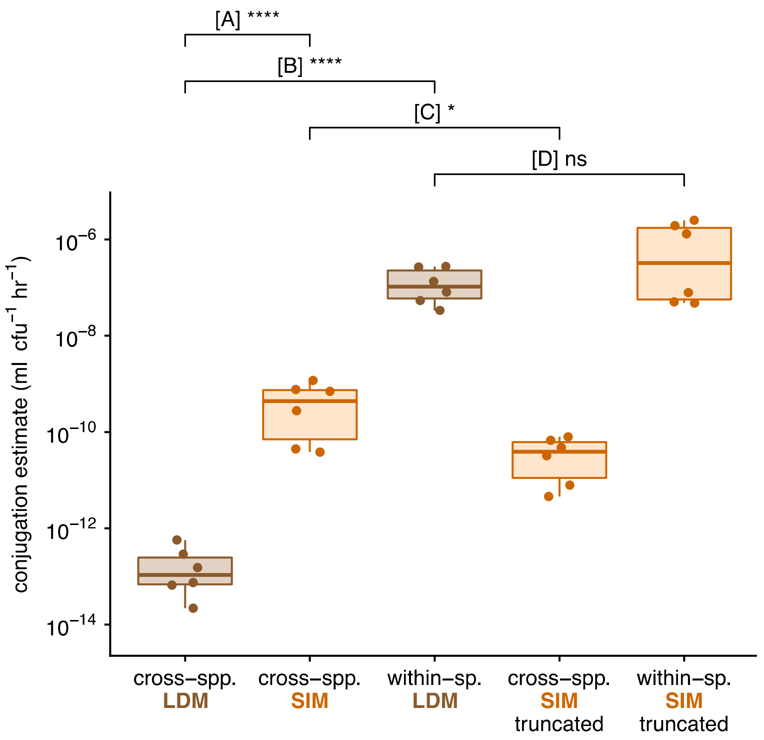

“**Figure 6 : Experimental estimates for cross-species and within-species conjugation rates.** Each box summarizes six replicate estimates by the LDM, SIM or truncated SIM approach, where each data point corresponds to a replicate. We note each of these estimates involved a correction (see Materials and Methods), but the same patterns hold for uncorrected values. [A] compares the LDM and standard SIM approach for a cross-species mating (between *K. pneumoniae* and *E. coli*). [B] compares the cross- and within-species mating using the LDM approach. [C] compares the standard and truncated SIM approach for a cross-species mating. [D] compares the LDM and truncated SIM approach for a within-species mating. The asterisks indicate statistical significance by a t-test (one, three and four asterisks convey p-values in the following ranges: $0.01 < p < 0.05$, $0.0001 < p < 0.001$ and $p < 0.0001$, respectively).”

We also made the following changes (highlighted words) to the relevant methods (lines 668 – 671):

“This protocol was repeated six times alongside the LDM replicates. For the within-species mating, the SIM method was executed as outlined above for the cross-species mating, but only the truncated SIM method was conducted using a 3-hour incubation period.”

-- Specific comments: --

a. The abstract and significance statement do not do the rest of the paper justice. Specifically, the significance of this manuscript to me seems more in the stochastic treatment of conjugation than in showing that commonly used methods are biased. Further, the results apply to all plasmids, not just those that carry antibiotic resistance.

We are grateful to the reviewer for sharing their perspective here. We have reworked the abstract to emphasize how our stochastic-based method deviates from previous deterministic approaches, and we have rewritten the significance statement to echo the same emphasis (and simultaneously underline that plasmids with antibiotic resistance genes are just one category of plasmids of interest—albeit one that is particularly relevant from a public health perspective). The abstract and significance statement are copied below with the relevant portions highlighted (lines 33 – 36):

“**Abstract**

To increase our basic understanding of the ecology and evolution of conjugative plasmids, we need a reliable estimate of their rate of transfer between bacterial cells. Current assays to measure transfer rate are based on deterministic modeling frameworks. However, some cell numbers in these assays can be very small, making estimates that rely on these numbers prone to noise. Here we take a different approach to estimate plasmid transfer rate, which explicitly embraces this noise. Inspired by the classic fluctuation analysis of Luria and Delbrück, our method is grounded in a stochastic modeling framework. In addition to capturing the random nature of plasmid conjugation, our new methodology, the Luria-Delbrück method (‘LDM’), can be used on a diverse set of bacterial systems, including cases for which current approaches are inaccurate. A notable example involves plasmid transfer between different strains or species where the rate that one type of cell donates the plasmid is not equal to the rate at which the other cell type donates. Asymmetry in these rates has the potential to bias or constrain current transfer estimates, thereby limiting our capabilities for estimating transfer in microbial communities. In contrast, the LDM overcomes obstacles of traditional methods by avoiding restrictive assumptions about growth and transfer rates for each population within the assay. Using stochastic simulations and experiments, we show that the LDM has high accuracy and precision for estimation of transfer rates compared to the most widely used methods, which can produce estimates that differ from the LDM estimate by orders of magnitude.

Significance Statement

Conjugative plasmids play significant roles in the dynamics of microbial communities. Conjugation, the horizontal transfer of plasmids from one cell to another, is a common means of spread for genes of ecological significance, including those encoding antibiotic resistance. For both public health modeling and a basic understanding of microbial population biology, accurate estimates of the rate of plasmid transfer are of great consequence. Widely used methods assume the process of conjugation is deterministic and, under certain conditions, lead to biased estimates that deviate from true values by several orders of magnitude. Therefore, we developed a new approach, inspired by the classic fluctuation analysis of Luria and Delbrück, which treats plasmid transfer as a random process. Our Luria-Delbrück method is straightforward to implement in the laboratory and can accurately estimate the rate of plasmid conjugation for different bacterial systems under a wide variety of conditions."

b. Fig 3:
- Panel A: the placement of the label "events occuring after t*" seems to suggest these are the only events occuring after t*. Perhaps the authors could move the label to above all lines/types of events, or reword to "additional events occuring after t*"
- Panel C vs E: related to the general comment above, this seems an "unfair" comparison of both methods since these are not 10'000 independent estimates of the LDM. Line 744: I would perhaps rephrase that these are calculated "After" instead of "for" different incubation periods.

We thank the reviewer for pointing this out. We changed the label in Figure 3 to 'additional events after t*' as the reviewer suggested. To clarify panel c and e, we proceeded with two changes. First, we made the label in panel c and e generic (changed to 'calculate SIM' and 'calculate LDM', respectively). Second, we made the following highlighted word changes to the relevant sentence in the figure legend to clarify the details (lines 767 – 799):

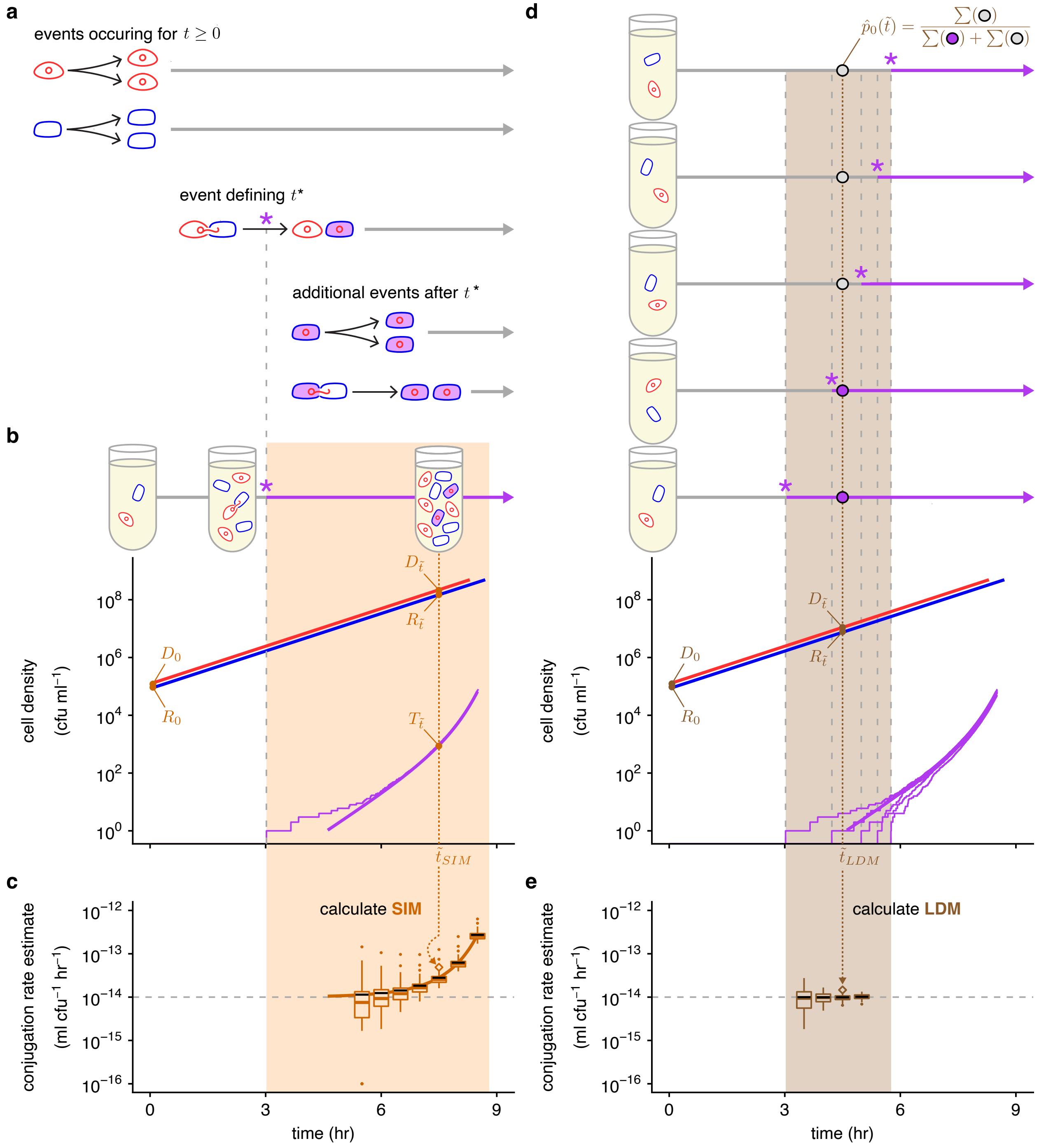

"Figure 3 : Overview of stochastic simulation framework and the effects of incubation time on estimating the conjugation rate. (a) The mating assay starts ($t = 0$) with donors and recipients and their populations increase over time. At a critical time (t^*, marked by a purple asterisk), the first transconjugant cell is generated through a conjugation event between a donor and recipient. After t^*, all possible growth and conjugation events can occur (including transconjugant division and conjugation). (b) A stochastic simulation of the equations [1]-[3] shows the donor, recipient, and transconjugant densities (red, blue, and purple thin trajectories, respectively) increasing over time. The deterministic numerical solution of the same equations and parameter settings from Figure 1b is shown for reference (thick lines). We note that for large densities, the stochastic and deterministic trajectories are closely aligned (i.e., the thick red and blue lines are overlaying their thin counterparts). After a specified incubation time ($\tilde{t}_{\text{SIM}}$, dotted orange line), we measure the densities of the three populations (orange $D_{\tilde{t}}$, $R_{\tilde{t}}$, and $T_{\tilde{t}}$), which can be used to calculate the

(c) SIM estimate. (d) Multiple mating assays are needed for the LDM estimate. Here, five stochastic simulations are shown, which display variation in t^*. At a specified incubation time ($\tilde{t}_{\mathrm{LDM}}$, dotted brown line), we determine the number of assay cultures with transconjugants (purple circles, where for a relevant culture $i,\ t_i^* < \tilde{t}_{\mathrm{LDM}}$) and without (gray circles, where for a relevant culture $j,\ t_j^* > \tilde{t}_{\mathrm{LDM}}$). These numbers are used to calculate $\hat{p}_0(\tilde{t})$, which, along with the donor and recipient densities (brown D_0, R_0, $D_{\tilde{t}}$ and $R_{\tilde{t}}$) are used for the (e) LDM estimate. The SIM (part c) and LDM (part e) estimates are calculated after different incubation times, where the $\tilde{t}_{\mathrm{SIM}}$ (part b) and $\tilde{t}_{\mathrm{LDM}}$ (part d) are indicated with orange and brown dotted arrows, respectively. The simulated trajectories in parts b and d would correspond to a single SIM or LDM estimate (the diamond points where the arrows terminate). The light orange and brown backgrounds indicate the range of incubation times giving a finite non-zero estimate of donor conjugation rate for the stochastic runs illustrated in parts b and d. In parts c and e, each box represents the distribution from 100 estimates of the donor conjugation rate for a given $\tilde{t}$, spanning from the 25th to 75th percentile. Given the log y-axis, the zero estimates are placed at the bottom of the y-axis range. The whiskers (i.e., vertical lines connected to the box) contain 1.5 times the interquartile range with the caveat that the whiskers were always constrained to the range of the data. The colored line in the box indicates the median. The solid black line indicates the mean. Parameter values are identical to Figure 1b and used throughout."

c. Line 241-242: it may help readers to reiterate that this intuition only holds early on during the mutational dynamics, when the R population is not too affected by transformation into transconjugants yet.

We thank the reviewer for the suggestion. We thought we could make the connection clearer by emphasizing a similarity in the structural set-up of our model and the Luria-Delbrück model, in line with the reviewer's focus. We have added the following to this section (lines 251 – 260) where the most relevant portion is highlighted:

"Comparing equation [12] to equation [11], conjugation can be thought of as a mutation process with initial wild-type population size $D_0 R_0$ that grows at rate $\psi_D + \psi_R$. The structural similarity of the estimates is grounded in a structural similarity of the underlying models; indeed, some of the same assumptions that apply to the mutation process modeled by Luria and Delbrück also apply to the conjugation process modeled here. For instance, the loss of recipients due to plasmid transfer is ignored in the recipient dynamics of the conjugation model (equation [6]) in the same way that the loss of wildtype cells due to mutation is ignored in the wildtype cell dynamics of the mutation model (equation [3.1] in SI Section 3), which tends to be a safe assumption when growth greatly outpaces transformation."

d. Line 258-259: this statement is quite simplified and ignores the fact that the SIM also requires the population sizes to be large enough to be detectable.

We agree that this could be made clearer. Thus, we have made the following highlighted word changes to the relevant sentence (lines 285 – 289):

"In the laboratory, the standard time point ($\tilde{t}$) used for the SIM estimate is 24 hours, however, a truncated assay ($\tilde{t} < 24$) also produces a non-zero estimate of the conjugation rate as long as the incubation time is greater than t^* (the orange region of Figure3b and c) and the density of transconjugants is large enough to be detected."

e. Line 260-269: At this point in the text it was not clear how many wells/simulations are needed for the maximum likelihood estimate of p0. Some info on this can be gleaned from SI Fig. 10, but it would be helpful to know how many wells are necessary for the LDM estimates to be accurate (like the convergence). Similarly, it would be good to know whether this depends on how close p0(t) is to 0 or 1 (how sensitive is the LDM to the statement in line 316-318).

We thank the reviewer for raising this point. We thought this question warranted further simulation, which we have added to the end of SI Section 8. We do find that slight bias can enter for small numbers of populations (which we can explain via Jensen's inequality). However, for the number of wells we are using (in the range between 50 and 100) the estimate appears to be accurate with low variance. In the new figure, we explicitly look at how variance changes with both the number of populations/wells and the assay time (which will affect the value of $p_0(\tilde{t})$). We include the new text and figure here for the convenience of the reviewer:

As illustrated in SI Figure 10, the variance in the LDM estimate changes with the number of populations (W). How does this number affect the variance in the LDM estimate? Here we use simulations to further explore this question. In SI Figure 11a, we present the variance of LDM estimates as a function of assay time ($\tilde{t}$) and the number of populations (W). Generally, as the number of populations decreases or as the boundaries of the time interval are approached (where nearly none or all of the populations have transconjugants) the variance in the LDM estimate rises. The exception seems to be for times that are very long, but the low variance is likely a result of having many infinite estimates that are not included in the estimate variance (SI Figure 11b). Both infinite estimates (SI Fig. 11b) and zero estimates (SI Fig. 11c) are more likely as the number of populations decreases; in other words, the interval of assay times producing non-zero finite estimates increases with the number of populations. Generally, the greater the number of populations and the more intermediate the assay time (e.g., where approximately half of the populations have transconjugants), the lower the variance.

Suppose an experimenter is considering some number of wells (populations) and wants to decide how many estimates to produce. For instance, with 500 wells, the experimenter could decide to run a single LDM assay and obtain a single estimate (with $\mathrm{W} = 500$) or perhaps instead could run 5 assays (with $\mathrm{W} = 100$), 10 assays (with $\mathrm{W} = 50$), 50 assays (with $\mathrm{W} = 10$) or 100 assays (with $\mathrm{W} = 5$) for 5, 10, 50, and 100 estimates, respectively. Does it make a difference to the precision or accuracy to split or lump wells? Here we explore this question through simulation. How do we compare different partitions of wells? Let us consider some total number of wells, call this W^*, and consider some factor of W^*, which we will call W'; i.e., $\mathrm{W}^*/\mathrm{W}' = n$, where n is an integer. Here we will compare a single estimate with W^* wells with the mean of n estimates that each use W' wells. Thus, for SI Figure 11d, each point for $\mathrm{W} = 500$ is a single estimate, where each point for $\mathrm{W} = 5$, $\mathrm{W} = 10$, $\mathrm{W} = 50$, and $\mathrm{W} = 100$ is the mean of 100, 50, 10, and 5 estimates, respectively. With these comparisons in mind, we see two slight effects of different partitioning patterns. First, the variance is a bit higher for the single estimate coming from the largest number of wells. We attribute this shift to the fact that other quantities involved in the estimate (e.g., density of donors and recipients) are only being computed once for each point for $\mathrm{W} = 500$ in Fig. SI 11d, whereas these quantities are being computed multiple times for smaller W values, such that anomalous values would tend to get muted as the estimates were averaged. The second effect is a more notable one. We see that as the number of wells per estimate goes down, slight inaccuracies in the estimate start to occur. Why does this happen?

To answer this question, let us consider the LDM estimate:

$$\gamma_D = -\ln p_0(\tilde{t})\left(\frac{\psi_D + \psi_R}{D_0 R_0\left(e^{(\psi_D+\psi_R)\tilde{t}} - 1\right)}\right)$$

The main thing that will be affected by the number of populations is $p_0(\tilde{t})$. Specifically, as W decreases, the variance in the fraction of populations without transconjugants increases. Suppose that we have n LDM estimates under consideration, and for each one a value $\hat{p}_0(\tilde{t})$ is needed. Here we define:

$$\overline{\hat{p}_0(\tilde{t})} = \frac{\sum_{i=1}^{n} \hat{p}_{0,i}(\tilde{t})}{n},$$

where $\hat{p}_{0,i}(\tilde{t})$ is the fraction of populations without transconjugants for the ith estimate. Now, by Jensen's inequality, we have:

$$-\ln\left\{\frac{\sum_{i=1}^{n} \hat{p}_{0,i}(\tilde{t})}{n}\right\}\left(\frac{\psi_D + \psi_R}{D_0 R_0\left(e^{(\psi_D+\psi_R)\tilde{t}} - 1\right)}\right) < \frac{1}{n}\sum_{i=1}^{n} -\ln \hat{p}_{0,i}(\tilde{t})\left(\frac{\psi_D + \psi_R}{D_0 R_0\left(e^{(\psi_D+\psi_R)\tilde{t}} - 1\right)}\right)$$

$$-\ln \overline{\hat{p}_0(\tilde{t})}\left(\frac{\psi_D + \psi_R}{D_0 R_0\left(e^{(\psi_D+\psi_R)\tilde{t}} - 1\right)}\right) < \frac{1}{n}\sum_{i=1}^{n} -\ln \hat{p}_{0,i}(\tilde{t})\left(\frac{\psi_D + \psi_R}{D_0 R_0\left(e^{(\psi_D+\psi_R)\tilde{t}} - 1\right)}\right)$$

As W gets large, the value $\hat{p}_0(\tilde{t})$ is close to $\overline{\hat{p}_0(\tilde{t})}$ for smaller W values. Thus, using the terminology from above:

$$\gamma_D[\mathrm{W}^*] < \frac{1}{n}\sum_{i=1}^{n} \gamma_D[\mathrm{W'}_i],$$

where $\gamma_D[\mathrm{W}^*]$ is the conjugation rate for the largest number of wells (W^*), and $\gamma_D[\mathrm{W'}_i]$ is the conjugation rate for the ith assay using a smaller number of wells ($\mathrm{W'}$). Thus, we see that as we partition wells into smaller numbers per estimate, the mean estimate will rise, which is what we see in SI Figure 11d. As a consequence, we recommend a reasonably large number of wells in the LDM assay. A number between 50 and 100 appears sufficient to avoid inaccuracy and is also convenient when using a microtiter plate format for populations.

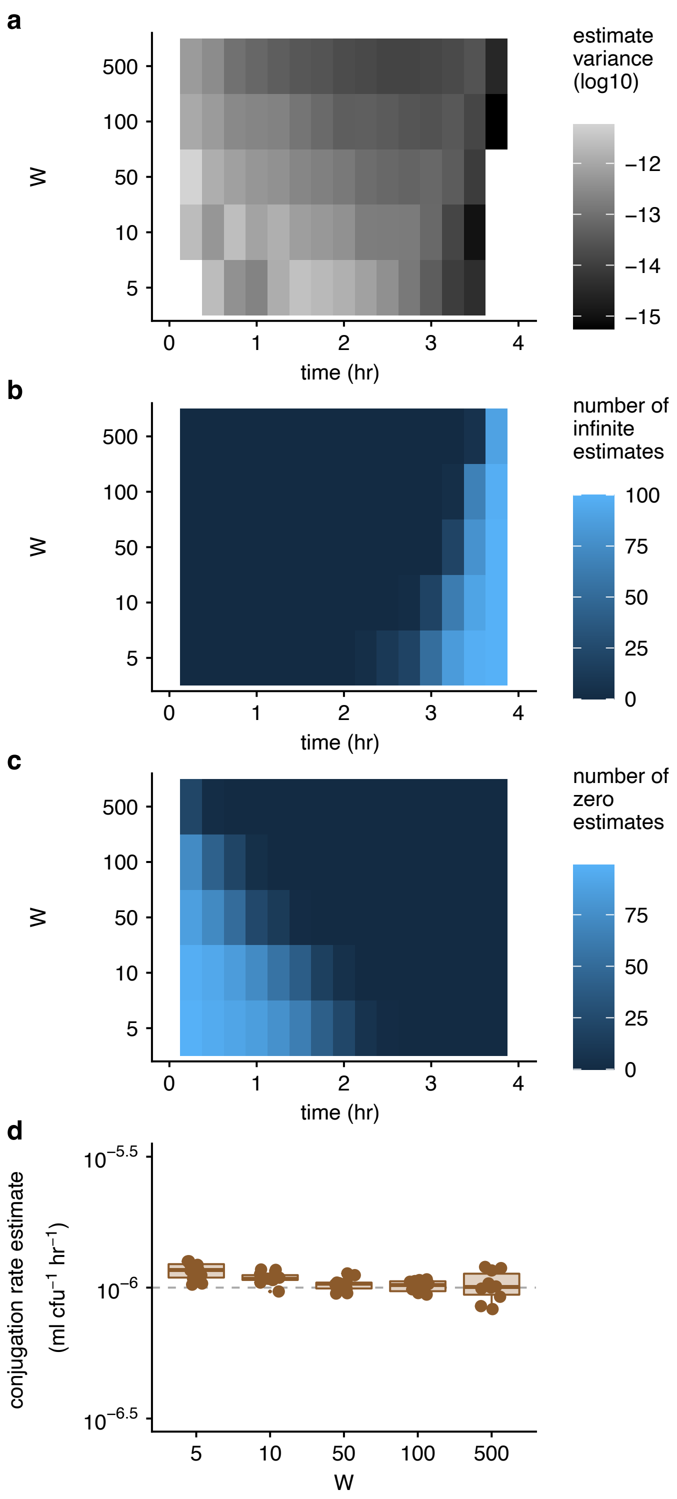

SI Figure 11: The variance of LDM estimates using stochastic simulation. Different number of populations (W) are used for the LDM estimates, as indicated. The parameters used here are the same baseline parameters in SI Figure 1 which were $\psi_D = \psi_R = \psi_T = 1$, and $\gamma_D = \gamma_T = 10^{-6}$. The dynamic variables were initialized with $D_0 = R_0 = 10^2$ and $T_0 = 0$. (a) The variation of each estimate is given at 15-minute intervals where more than 1 out of the 100 calculated estimates produced a finite non-zero value. We ignore infinite estimates in the calculation of the variance. (b) The number of estimates with an infinite

value out of the 100 calculated. (c) The number of estimates with a zero value out of the 100 calculated. (d) A total of 500 populations is partitioned in different ways—split into 100 groups of 5 populations (W=5), 50 groups of 10 populations (W=10), 10 groups of 50 populations (W=50), 5 groups of 100 populations (W=100), or a single group of 500 populations (W=500). Each plotted point is the mean conjugation rate of the rates calculated for each group (where the number of populations within each group vary as indicated by the W value). Each partitioning was run 10 times.

f. Line 292-294: This description is unclear and would benefit from stating more explicitly that the Gillespie algorithm gets slow when population sizes are large (i.e. that the difference between both parameter sets is in D0, R0 and gammaD).

We agree that more detail would help clarify things. Thus, we have made the following highlighted word changes to the relevant sentence (lines 322 – 325):

"Given the large number of simulations for these sweeps, we chose parameter values (i.e., D_0, R_0, and γ_D) outside of typical values reported in the literature to reduce the computational burden of the Gillespie algorithm, which occurs when populations sizes become very large."

g. Cross-species case study (line 366): when and how was the growth rate of the individual strains determined?

We thank the reviewer for pointing out that this was not clearly linked to our supplemental information. Thus, we have made the following highlighted words to a relevant sentence in the third paragraph in the 'Cross-species case study' section (lines 409 – 412):

"Second, our growth rate assays (conducted separately from the transfer estimate protocols; see SI section 6b) revealed our cell types have different growth rates (SI Figure 8), thus violating the SIM assumptions."

Line 358-365: The authors did not test that the resource dependence is the same for conjugation and growth. As such, this paragraph seems to suggest a generality that doesn't hold, and the SIM estimate may be unnecessarily bad by running the assay over 24 instead of shorter (e.g. 6 hours).

Our apologies for the lack of clarity. We did not mean to suggest that growth and conjugation is proportional in our experimental system. We meant to explain to the reader that this is an assumption embedded in the SIM approach and is an assumption of particular relevance for the 24-hour (standard) SIM protocol (as clearly growth rates decline as a batch culture enters stationary phase). Except for the original Simonsen et al. study, many researchers using the SIM estimate do not check this (or other) assumptions before calculating the metric using the standard assay. Our aim was to show that a failure to confirm underlying assumptions can lead to problems (although we focus more heavily on the assumption of transfer rate identicality). To clarify our intentions, we added the following highlighted sentences to the appropriate section (lines 396 – 402):

"If conjugation rates decrease with resources in a similar fashion and the parametric identicality assumptions hold, the SIM estimate can be used over a full-day incubation. Despite the restrictive underlying assumptions, it is not uncommon for researchers to estimate plasmid transfer rates using the 24-hour SIM protocol without experimental validation of assumptions. We proceeded with the standard SIM protocol in order to allow a comparison between this popular estimate and our new estimate (resulting from the LDM protocol, which does not rest on the same assumptions)."

h. Line 494: Which violations of model assumptions is meant for the Dewar et al study here?

We apologize for the confusion. In this case, the Dewar *et al*. investigation published in 2021 in Nature Ecology & Evolution used conjugation rates as a key parameter in their modeling study. This reference was meant to emphasize the current urgency for estimating conjugation rates that the authors of this study point out at several places in their manuscript. For example, the Dewar *et al*. study states "A caveat here is that our estimates of transfer rates across different types of plasmid is relative and it would be very useful to obtain quantitative estimates of transfer rates." To clarify, we made the following highlighted adjustments to the relevant sentence (lines 536 – 538):

"Given recent interest in quantitative estimates of conjugation rate (29) and the impacts of model assumption violations (1, 2), there has been a matching interest in altering conjugation protocols such that bias is minimized when violations apply."

i. Line 530-536: I do not understand this section about the functional response and the conditionality of different methods. Could the authors explain their argument in different words?

We thank the reviewer for communicating potential for confusion here. We decided to remove these two sentences-- as they are not necessary for the main point of the paragraph, and elaboration might end up breaking the flow of the paragraph. However, we will offer an explanation in this response letter.

The model underlying the SIM approach does not assume that the conjugation rate is constant. Similar to growth rate, the model assumes that conjugation rate changes as a Monod function of resource concentration (C):

$$\gamma(C) = \frac{\gamma_{max} C}{Q + C},$$

where Q is the half saturation constant and γ_{max} is the asymptotic conjugation rate as $C \to \infty$. Thus, the model incorporates exactly how conjugation rate changes with environmental conditions (resources). Thus, there is a core conditionality built into the model—the conjugation rate is (an increasing and saturating) function of resource level. The SIM provides an estimate for a parameter, γ_{max}, of this functional response. However, there is then the additional issue of whether this functional response is itself appropriate (e.g., some plasmids continue to transfer under stationary phase conditions, where resource levels are low).

In case the reviewer is interested, we offer a slight generalization for the SIM estimate in our GitHub Appendix II. Specifically, the SIM estimate can be used if parametric homogeneity of growth and transfer rates applies across strains, and if growth rate has a functional response $\psi(C) = \psi^{\blacksquare} g(C)$ and conjugation rate has a functional response $\gamma(C) = \gamma^{\blacksquare} g(C)$. The quantities $\psi^{\blacksquare}$ and $\gamma^{\blacksquare}$ are constant parameters and $g(C)$ is some shared function of resource concentration. In this case, the SIM estimate captures the constant parameter $\gamma^{\blacksquare}$ (although a researcher would have to also estimate $\psi^{\blacksquare}$). The point is that other functional responses are possible (i.e., the SIM estimate does not require Monod functions), but the constraint that growth and transfer respond (functionally) similarly to resource level remains.

j. Line 591: The number of 84 wells does not seem to match the number of black bordered wells in Fig. 5 (87 wells).

We thank the reviewer for pointing this out. To clarify where the 84 wells is coming from, we have made the following highlighted word changes to the relevant sentence (lines 626 – 632):

"The exponentially growing cultures were diluted by a factor specific to the donor-recipient pair (SI section 6e), mixed at equal volumes, and dispensed into 84 wells of a deep-well microtiter plate at 100 µl per well (Figure 5a black-bordered wells in rows 2 - 8, these wells were the co-cultures used to estimate $p_0(\tilde{t})$). In an additional 3 wells (Figure 5a black-bordered wells in top row), 130 µl (per well) of the mixture was

dispensed and immediately 30 μl was removed to determine the initial densities (D_0 and R_0) via selective plating."

k. Line 638-646: I would like to see this discussion on establishment probability (+ perhaps stochasticity in pipetting etc.) more prominently discussed in the main text. In particular I would assume the effect of this stochastic extinction is different for the SIM estimate vs. the LDM, but it would be good to test this with simulations.

In response to the reviewer point #1 (our new SI Section 9) we have simulated the effects of different establishment probabilities (and dilution/plating random effects) on both the LDM and SIM estimates. As shown in SI Fig. 12b, there do seem to be greater impacts on the SIM estimate than the LDM estimate.

We have also added an additional reference to the establishment probability SI sections at the end of our first results section in the main text (with the relevant bit highlighted from lines 263 – 272):

"Lastly, as in the original Luria-Delbrück model, we focus on a pure 'birth' process (e.g., once mutants or transconjugants are generated, their numbers do not decrease). In our supplemental sections we explore the impacts of violations to some of these assumptions (e.g., the negligible impact of segregational loss in SI Section 4d, and how to correct for an effective loss in transconjugant cell numbers due to a failure of small numbers of transconjugants to establish under experimental conditions in SI Sections 6d and 7). Given the connections between modeling frameworks and estimate structures, we label the expression in equation [11] as the LDM estimate for donor conjugation rate, where LDM stands for 'Luria-Delbrück Method.' "

l. Line 656: It would be helpful to mention this information on the number of populations used to estimate the LDM (vs. other methods) also in the main text (e.g., around line 275) and/or in the figure captions.

We agree with the reviewer that this detail could appear earlier in the text. Thus, we have made the following highlighted word changes to the suggested sentence (lines 290 – 294):

"While the SIM estimate uses the density of transconjugants ($T_{\tilde{t}}$), the LDM equation instead involves $p_0(\tilde{t})$, the probability that a population has no transconjugants at the end of the assay. A maximum likelihood estimate for this probability (hereafter $\hat{p}_0(\tilde{t})$) is obtained by calculating the fraction of populations that have no transconjugants (e.g., 100 parallel simulations were used) at the specific incubation time $\tilde{t}$ (top of Figure 3d)."

m. Fig. 4: why did the runs result in SIM estimates of zero? Was the recipient population 0 in these simulations?

This is a very good question. The SIM estimates are zero due to a zero transconjugant density at the chosen incubation time. To make a fair comparison across the different parametric combinations shown in Figure 4, we calculated the SIM where on average there is 50 transconjugants. When the transconjugant conjugation rate is so much higher than the donor conjugation rate, the time where there is on average 50 transconjugants shifts to an earlier point in time in comparison to a scenario where the donor conjugation rate and transconjugant conjugation rate are closer together. At these earlier time points, some runs have zero transconjugants (leading to SIM estimates of zero). To provide more information about the different incubation times used in each scenario and the source of the zero values, we have made the following highlighted word changes and provided a supplementary table:

Lines 711 – 714:
"To compare across various parameter settings (Figure 4), a single incubation time was chosen per parameter set and type of estimate (See SI Table 5 for the incubation times used)."

Figure 4 legend (lines 812 – 816):
"For the 10^{-2} transconjugant conjugation rate, many of the runs resulted in SIM estimates of zero due to zero transconjugants at the specific incubation time; therefore, the median (colored line) and the box are placed at the bottom of the plot (given that the y-axis is on a log scale)."

SI Table 5: Specific incubation times ($\tilde{t}$) used in stochastic simulations to compare across parameter settings. Each row lists the relevant figure and the corresponding x-axis value. Time is given in hours. For each parameter setting, the incubation time $\tilde{t}$ for the LDM estimate is set to the average t^*, and for the SIM estimate is given by the time point for which an average of 50 transconjugants is reached.

**Figure**	**x-axis value**	$\tilde{t}_{\mathbf{LDM}}$	$\tilde{t}_{\mathbf{SIM}}$
Figure 4, SI Figure 1a	0.0625	4.34	7.89
Figure 4, SI Figure 1a	0.125	4.11	7.49
Figure 4, SI Figure 1a	0.25	3.7	6.78
Figure 4, SI Figure 1a	0.5	3.1	5.67
Figure 4, SI Figure 1a	1	2.35	4.27
Figure 4, SI Figure 1a	2	1.61	2.86
Figure 4, SI Figure 1a	4	1.01	1.74
Figure 4, SI Figure 1a	8	0.6	1
Figure 4, SI Figure 3	1×10^9	2.35	4.27
Figure 4, SI Figure 3	1×10^8	2.35	4.27
Figure 4, SI Figure 3	1×10^7	2.35	4.27
Figure 4, SI Figure 3	1×10^6	2.35	4.27
Figure 4, SI Figure 3	1×10^5	2.35	4.25
Figure 4, SI Figure 3	1×10^4	2.33	4.11
Figure 4, SI Figure 3	1×10^3	2.16	3.4
Figure 4, SI Figure 3	1×10^2	1.44	2.02
SI Figure 1b	0.0625	4.35	8.18
SI Figure 1b	0.125	4.11	7.66
SI Figure 1b	0.25	3.71	6.85
SI Figure 1b	0.5	3.1	5.69
SI Figure 1b	1	2.35	4.27
SI Figure 1b	2	1.61	2.86
SI Figure 1b	4	1.01	1.74
SI Figure 1b	8	0.6	0.99
SI Figure 2a	0.0625	3.3	6.4
SI Figure 2a	0.125	3.22	6.23
SI Figure 2a	0.25	3.07	5.89
SI Figure 2a	0.5	2.8	5.26
SI Figure 2a	1	2.35	4.27

SI Figure 2a	2	1.78	3.07
SI Figure 2a	4	1.2	1.99
SI Figure 2a	8	0.74	1.15
SI Figure 2b	0.0625	3.31	6.45
SI Figure 2b	0.125	3.23	6.27
SI Figure 2b	0.25	3.07	5.92
SI Figure 2b	0.5	2.8	5.27
SI Figure 2b	1	2.35	4.27
SI Figure 2b	2	1.78	3.07
SI Figure 2b	4	1.2	1.97
SI Figure 2b	8	0.74	1.15
SI Figure 2c	0.0625	2.64	4.59
SI Figure 2c	0.125	2.62	4.57
SI Figure 2c	0.25	2.59	4.54
SI Figure 2c	0.5	2.52	4.46
SI Figure 2c	1	2.35	4.27
SI Figure 2c	2	1.97	3.62
SI Figure 2c	4	1.34	2.31
SI Figure 2c	8	0.8	1.29
SI Figure 4	0.00001	2.35	4.27
SI Figure 4	0.0001	2.35	4.27
SI Figure 4	0.001	2.35	4.27
SI Figure 4	0.01	2.36	4.29
SI Figure 4	0.1	2.47	4.49

n. SI table 3: The text reads "The SIM model can incorporate resource-dependent growth and conjugation *because* (1) growth and transfer rates are homogeneous", but I would rather think this holds *if/when* these two conditions are given.

We thank the reviewer for catching this mistake. We have changed the footnote in SI Table 3 (SI line 121) to read:

“The SIM model can incorporate resource-dependent growth and conjugation if (1) growth and transfer rates are homogeneous and (2) the functional form for resource dependence is the same for growth and transfer.”

o. Github folder:
- I could not find the code that was used to produce the simulations that are shown in SI Fig. 1 (or Fig. 1 for that matter), only the code to plot those simulation results.
- Some readme pages contain comments that look like they were meant to be internal/private (e.g. https://github.com/livkosterlitz/LDM/tree/main/Figures), and others don't display well (https://github.com/livkosterlitz/LDM/tree/main/Simulations and https://github.com/livkosterlitz/LDM/tree/main/Supporting_data).

We apologize for the mistakes on the GitHub documentation. We have remedied the ReadMe pages in the repository. In addition, the code for generating all the simulation data and the supporting documentation can be found at https://github.com/livkosterlitz/LDM/tree/main/Simulations.

p. SI Fig 1-5:
- The TDR estimate is consistently a factor 10 smaller than the other estimates. Can the authors explain why?

This is an interesting question, but the answer is a bit involved.

In the GitHub appendices, we derive analytical expressions for the number of donors, recipients, and transconjugants under the ASM model assumptions (i.e., in which the recipient population is dominated by growth and the transconjugant population is dominated by growth and conjugation from donors). Specifically,

$$D_{\tilde{t}} = D_0 e^{\psi_D \tilde{t}},$$

$$R_{\tilde{t}} = R_0 e^{\psi_R \tilde{t}},$$

$$T_{\tilde{t}} = \frac{\gamma_D}{\psi_D + \psi_R - \psi_T} \{ D_0 R_0 e^{(\psi_D + \psi_R)\tilde{t}} - D_0 R_0 e^{\psi_T \tilde{t}} \}.$$

Thus, the TDR estimate can also be written analytically

$$\frac{T_{\tilde{t}}}{D_{\tilde{t}} R_{\tilde{t}} \tilde{t}} = \gamma_D \frac{1 - e^{-(\psi_D + \psi_R - \psi_T)\tilde{t}}}{(\psi_D + \psi_R - \psi_T)\tilde{t}}$$

The TDR estimate is accurate only when there is no population growth. As either each of ψ_D, ψ_R, and ψ_T approaches zero or as $\tilde{t}$ goes to zero, the TDR estimate becomes increasingly accurate. Letting $\alpha = (\psi_D + \psi_R - \psi_T)\tilde{t}$, we can show this mathematically, because all these conditions are encompassed by $\alpha \to 0$, and

$$\lim_{\alpha \to 0} \frac{T_{\tilde{t}}}{D_{\tilde{t}} R_{\tilde{t}} \tilde{t}} = \lim_{\alpha \to 0} \gamma_D \frac{1 - e^{-\alpha}}{\alpha}.$$

Using L'Hôpital's rule,

$$\lim_{\alpha \to 0} \frac{T_{\tilde{t}}}{D_{\tilde{t}} R_{\tilde{t}} \tilde{t}} = \lim_{\alpha \to 0} \gamma_D \frac{e^{-\alpha}}{1} = \gamma_D$$

However, $\alpha = (\psi_D + \psi_R - \psi_T)\tilde{t}$ may be non-zero, and the TDR estimate will deviate from the donor conjugation rate (γ_D).

In the simulations in the Supplementary Information, the baseline parameters are $\psi_D = \psi_R = \psi_T = 1$. With $\tilde{t} \approx 4.25$,

$$\frac{T_{\tilde{t}}}{D_{\tilde{t}} R_{\tilde{t}} \tilde{t}} \approx 0.23\gamma_D,$$

such that the TDR estimate is a little shy of one order of magnitude below the actual donor conjugation rate. Many of the parameter values produce a factor $(1 - e^{-\alpha})/\alpha$ that is close to the baseline value, which is why the TDR estimate is about one order of magnitude in many of the plotted cases.

However, we can use our formula to explain the more unusual deviations too. For instance, consider $\psi_D = \psi_R = 1$ and $\psi_T = 8$. With $\tilde{t} \approx 1.3$,

$$\frac{T_{\tilde{t}}}{D_{\tilde{t}} R_{\tilde{t}} \tilde{t}} \approx 529 \gamma_D,$$

such that we see the TDR estimate is over two orders of magnitude greater than the actual donor conjugation rate (see the mean value for the rightmost transconjugant growth rate on the TDR plot in SI Figure 2c).

As long as the basic assumptions of the ASM hold, the expression:

$$\frac{T_{\tilde{t}}}{D_{\tilde{t}} R_{\tilde{t}} \tilde{t}} = \gamma_D \frac{1 - e^{-(\psi_D + \psi_R - \psi_T)\tilde{t}}}{(\psi_D + \psi_R - \psi_T)\tilde{t}}$$

Gives a good accounting of how the TDR deviates from the donor conjugation rate (γ_D), namely by the factor:

$$\frac{1 - e^{-(\psi_D + \psi_R - \psi_T)\tilde{t}}}{(\psi_D + \psi_R - \psi_T)\tilde{t}}.$$

- Some legends read "Zero estimates were set to 10^-9": why were these 0? One could argue that in this scenario and experimenter would clearly see something is wrong, and would redo the experiment for a different incubation period / initial pop sizes.

As discussed under comment ‘m’, additional text and an SI table were added to address potential confusion surrounding incubation times and the source of zero estimates.

- Do I understand the Material and Methods correctly that the incubation period was changed for each method depending on when a certain # tranconjugants were reached, i.e. dependent on the transconjugant growth rate + the conjugation rate (e.g. separately for each x-position in Fig S1)?

Yes, the reviewer is correct. We thank the reviewer for comments regarding potential confusion regarding chosen incubation times for analyzing the stochastic simulations. As discussed under comment ‘m’, additional text and SI table 5 were added to provide more information about incubation times.

- The critical time threshold of the ASM is mentioned only for SI Fig. 3 (Line 386-390) but it looks like this may have been a factor already in SI Fig. 2.

We agree with the reviewer that the ASM disclaimer appeared in the wrong place in the supplement. Thus, we have moved the relevant highlighted text to SI section 4a, the first portion of the ‘Extended Simulation Results’ in the supplemental material where we also include the TDR disclaimer (SI lines 323 – 334):

“The incubation time selection criteria used for the SIM estimate was also used for the TDR and ASM estimates (see Materials and Methods and SI Table 5). However, given that all our simulated populations *increase in size* over the incubation time, a fundamental assumption of the TDR approach is broken for all

the runs (i.e., no change in the density due to growth). The TDR estimate was included to be comprehensive (and illustrate that violation of the no growth assumption leads to systemic bias). Also, we note that we calculated the ASM metric in all scenarios and that in some cases the incubation time $\tilde{t}$ passed the critical time threshold (t_{crit}) where the ASM assumptions break down (see SI section 1a). The ASM estimate was included in all scenarios to be comprehensive and illustrate that implementing the assay after t_{crit} can lead to bias. Given that the chosen incubation time $\tilde{t}$ to evaluate these simulations is early, it highlights that for some parametric combinations proper implementation of the ASM metric is not possible."

q. Line 376: It was insufficiently clear that this reference to SI section 4 refers to sections 4fgh, and within those sections not so clear what the assumptions and outcomes are (+ how they relate to the cross-species conjugation experiment).

We agree with the reviewer that we could have been clearer. We updated the main text reference to be the relevant SI figure. In addition, we added the highlighted text to the relevant section in the supplement to provide additional information.

Lines 412 – 413 in the main text:
"While simulations show there is an effect of these inequalities, the effect size is insufficient to explain the observed difference in comparison A (SI Figure 6)."

SI lines 478 – 501 in the supplement:
'Here we used equations [4.5]-[4.12] that incorporate batch culture dynamics to simulate the cross-species case study with the experimentally measured parameters to investigate the incongruency observed between the SIM and LDM estimates in Figure 6. Most of the parameters were from the average of six experiments (D_0 = 1.17 x 10^{5}, R_0 = 3.33 x 10^{4}, ψ_D = 1.91, ψ_R = 1.47, ψ_T = 1.48, γ_{DR} = 1.96 x 10^{-13}, and γ_{TR} = 1.96 x 10^{-7}) with the remaining parameters informed by the 24 hour densities as to mimic the batch culture conditions of the experiment (C_0 = 4.41 x 10^{9}, Q = 1 x 10^{7}, and e = 1). We used the numerical solution to calculate the SIM estimate over time.

We compared the numerical solution to the actual experimental measurements from the cross-species experiments. The simulated density and conjugation estimate (SI Figure 6a solid lines) were similar to the average experimental densities and the experimental SIM estimate (SI Figure 6a circle data points). Thus, the experimental LDM estimates for the cross-species conjugation rates (γ_{DR} = 1.96 x 10^{-13}) and the within-species (γ_{TR} = 1.96 x 10^{-7}) along with the measured growth rates are sufficient to recapture a relatively inflated experimental SIM estimate. In contrast, a simulation with homogenous conjugation rates using either the cross- or within-species conjugation rate does not closely align with the experimental data (SI Figure 6b and c, respectively). These simulations also demonstrate that the heterogeneity in the measured growth rates is insufficient to produce the mismatch observed in the experimental data (SI Figure 6b and c). This was worth checking given that heterogeneity in growth rates violates a modeling assumption of the SIM approach. This adds further support that the parametric heterogeneity in the conjugation rates is the potential cause for the incongruency between the LDM and SIM estimates reported in Figure 6."

- How good is the assumption (SI Line 476/477) that conjugation and plasmid loss are Monod functions of the resource concentration?

The reviewer asks a good question. To our knowledge the functional dependence of conjugation rate and plasmid loss rate on resource concentration has not received a great deal of attention. Simonsen *et al.* (1990) did justify the Monod function assumption for the IncF plasmid used in their study (as plasmid transfer, similar to growth, halted in stationary phase). A case could be made that plasmid loss—given its dependence on cell division—might possess a rate that mirrors the functional form of the growth rate. However, we think it is likely that the assumption that conjugation rate is generally a Monod function will be violated. For instance, there are plasmids that continue to transfer even when cells are not growing (3); thus, if

resource depletion corresponds to zero growth, it does not always correspond to zero conjugation such that conjugation is not a standard Monod function.

We note that the derivation of the Simonsen *et al*. estimate does not require Monod functions, *per se*, but does require similarity between growth and conjugation as a function of resource such that the ratio of these rates remain constant across resource values. However, we think this assumption is also likely to be violated in specific systems.

We emphasize that the LDM estimate focuses on populations exhibiting constant growth over relatively short periods of time (in which resource levels are taken to be relatively constant) and it is assumed that conjugation rate is also constant over the assay. Other estimates (e.g., SIM and ASM) can be calculated over short periods where growth and conjugation rate are close to constant. In such cases, there is no assumption that conjugation rate has any particular functional dependence on resources. Indeed, we argue that executing the LDM multiple times across a resource gradient would be a way to explore the relationship between resource level and conjugation rate.

- Caption of Fig S6: have these significant deviations (Panel b/c) been tested statistically? Are the parameter estimates from the LDM?

We thank the reviewer for catching this. Our language was sloppy here. We were referring to the striking mismatch between the model and the data, but we did not perform statistical tests here. We have rephrased this legend replacing the word "significantly." The end of the legend for Fig S6 as the following highlighted word changes (lines 511 – 514) and now reads:

"(b) A scenario with homogenous low conjugation rates (γ_{DR} = γ_{TR} = 1.96 x 10^{-13}) deviates markedly from the experimental measurements. (c) A scenario with homogenous high conjugation rates (γ_{DR} = γ_{TR} = 1.96 x 10^{-7}) deviates substantially from the experimental measurements."

- Is Fig S7 repeated with the same parameters as Fig S6? The descriptive text is written as if this is general behavior of the SIM, but the depletion of the recipient pool seems parameter-specific?

We appreciate the reviewer pointing this out. The reviewer is correct that the same parameters used in Fig S6 are used in Fig S7. Also, we apologize for not properly emphasizing that the behavior in Fig S7 is parameter specific. We added the highlighted text to the relevant section in the supplement to clarify (lines 518 – 532):

"In this section, we explored a violation of a modeling assumption in the SIM approach by using a model variation where the functional form of growth rate and conjugation rate are not proportional. This is relevant given that there are plasmid systems that will readily violate this proportional assumption (e.g., IncP plasmids). Here, we assume that while growth rates follow the Monod equation, conjugation rates are not dependent on resource and remain constant after resources are depleted. We found that using this model and the same experimentally measured parameter values used in SI Figure 6 resulted in a higher SIM estimate at 24 hours (SI Figure 7a) compared to the scenario where conjugation rate is proportional to growth rate (SI Figure 7b). It is worth noting that by using this new model and these particular parameter values, the recipient pool is completely depleted which coincides with the SIM estimate no longer being a finite, positive value. This differs from SI Figure 7b where the SIM estimate hits an asymptote remaining at a finite, positive value. In this case, the recipient pool is not depleted because in this version of the model (SI section 4f) the conjugation rates approach zero as the resources are depleted."

Reviewer 2:

In this manuscript, Kosterlitz and colleagues propose an elegant and effective method for estimating plasmid conjugation rates. The key innovation, inspire by Luria and Delbrück's classic of (microbial) evolutionary genetics, is to use presence/absence of transconjugants across multiple cultures, rather than absolute counts from a single culture. Simulations and data convincingly show that the proposed method is more robust than existing approaches, and the authors provide extensive (re-)derivations enabling comparison with the state-of-the-art. Overall I thought this was an excellent piece of work and makes a valuable contribution to the field. The analyses are comprehensive, but clearly written and easy to follow even for a non-mathematician. Supplementary Information and Figures are excellent.

We sincerely thank the reviewer for these positive comments.

I do, however, have some suggestions/comments for consideration, which may improve things further.

1. In the proposed LDM approach, p[hat]_0(t[tilde]) is the probability of zero mutants at time t[tilde] and is estimated by establishing multiple cultures and counting the proportion of negatives. p[hat]_0(t[tilde]) is therefore an estimate, the accuracy of which will presumably improve with the number of cultures established. There is not much discussion/analysis of how the accuracy of the overall measurement will vary with the number of cultures. This has consequences for experimental design. While the recommended 96-well plate design is sensible, I wonder if there is a minimum number of cultures that is required for accurate calculation? Or is it worth investing more resources to run more cultures to improve accuracy? Is the proposed one-plate-per-measurement approach the most efficient?

We thank the reviewer for raising this question, which we felt warranted additional simulations. We have added the results to the end of SI Section 8, as we follow up on the issues of estimate variance discussed in that section. For small numbers of cultures (e.g., 5 or 10 wells) slight bias can enter into the LDM estimate, which we can explain via Jensen's inequality—see below). However, for the number of wells we are using (between 50 and 100) the estimate is accurate and precise. We also explore the effects of "partitioning" wells to achieve different numbers of estimates. Again, we find that partitioning so that the total number of populations per estimate is in the range of 50-100 wells appears to optimize accuracy and precision. We include the new text and figure here for the convenience of the reviewer:

As illustrated in SI Figure 10, the variance in the LDM estimate changes with the number of populations (W). How does this number affect the variance in the LDM estimate? Here we use simulations to further explore this question. In SI Figure 11a, we present the variance of LDM estimates as a function of assay time ($\tilde{t}$) and the number of populations (W). Generally, as the number of populations decreases or as the boundaries of the time interval are approached (where nearly none or all of the populations have transconjugants) the variance in the LDM estimate rises. The exception seems to be for times that are very long, but the low variance is likely a result of having many infinite estimates that are not included in the estimate variance (SI Figure 11b). Both infinite estimates (SI Fig. 11b) and zero estimates (SI Fig. 11c) are more likely as the number of populations decreases; in other words, the interval of assay times producing non-zero finite estimates increases with the number of populations. Generally, the greater the number of populations and the more intermediate the assay time (e.g., where approximately half of the populations have transconjugants), the lower the variance.

Suppose an experimenter is considering some number of wells (populations) and wants to decide how many estimates to produce. For instance, with 500 wells, the experimenter could decide to run a single LDM assay and obtain a single estimate (with W = 500) or perhaps instead could run 5 assays (with W = 100), 10 assays (with W = 50), 50 assays (with W = 10) or 100 assays (with W = 5) for 5, 10, 50, and 100 estimates, respectively. Does it make a difference to the precision or accuracy to split or lump wells? Here

we explore this question through simulation. How do we compare different partitions of wells? Let us consider some total number of wells, call this W^*, and consider some factor of W^*, which we will call W'; i.e., $W^*/W' = n$, where n is an integer. Here we will compare a single estimate with W^* wells with the mean of n estimates that each use W' wells. Thus, for SI Figure 11d, each point for $W = 500$ is a single estimate, where each point for $W = 5$, $W = 10$, $W = 50$, and $W = 100$ is the mean of 100, 50, 10, and 5 estimates, respectively. With these comparisons in mind, we see two slight effects of different partitioning patterns. First, the variance is a bit higher for the single estimate coming from the largest number of wells. We attribute this shift to the fact that other quantities involved in the estimate (e.g., density of donors and recipients) are only being computed once for each point for $W = 500$ in Fig. SI 11d, whereas these quantities are being computed multiple times for smaller W values, such that anomalous values would tend to get muted as the estimates were averaged. The second effect is a more notable one. We see that as the number of wells per estimate goes down, slight inaccuracies in the estimate start to occur. Why does this happen?

To answer this question, let us consider the LDM estimate:

$$\gamma_D = -\ln p_0(\tilde{t})\left(\frac{\psi_D + \psi_R}{D_0 R_0\left(e^{(\psi_D+\psi_R)\tilde{t}} - 1\right)}\right)$$

The main thing that will be affected by the number of populations is $p_0(\tilde{t})$. Specifically, as W decreases, the variance in the fraction of populations without transconjugants increases. Suppose that we have n LDM estimates under consideration, and for each one a value $\hat{p}_0(\tilde{t})$ is needed. Here we define:

$$\overline{\hat{p}_0(\tilde{t})} = \frac{\sum_{i=1}^{n} \hat{p}_{0,i}(\tilde{t})}{n},$$

where $\hat{p}_{0,i}(\tilde{t})$ is the fraction of populations without transconjugants for the ith estimate. Now, by Jensen's inequality, we have:

$$-\ln\left\{\frac{\sum_{i=1}^{n} \hat{p}_{0,i}(\tilde{t})}{n}\right\}\left(\frac{\psi_D + \psi_R}{D_0 R_0\left(e^{(\psi_D+\psi_R)\tilde{t}} - 1\right)}\right) < \frac{1}{n}\sum_{i=1}^{n} -\ln \hat{p}_{0,i}(\tilde{t})\left(\frac{\psi_D + \psi_R}{D_0 R_0\left(e^{(\psi_D+\psi_R)\tilde{t}} - 1\right)}\right)$$

$$-\ln \overline{\hat{p}_0(\tilde{t})}\left(\frac{\psi_D + \psi_R}{D_0 R_0\left(e^{(\psi_D+\psi_R)\tilde{t}} - 1\right)}\right) < \frac{1}{n}\sum_{i=1}^{n} -\ln \hat{p}_{0,i}(\tilde{t})\left(\frac{\psi_D + \psi_R}{D_0 R_0\left(e^{(\psi_D+\psi_R)\tilde{t}} - 1\right)}\right)$$

As W gets large, the value $\hat{p}_0(\tilde{t})$ is close to $\overline{\hat{p}_0(\tilde{t})}$ for smaller W values. Thus, using the terminology from above:

$$\gamma_D[W^*] < \frac{1}{n}\sum_{i=1}^{n} \gamma_D[W'_i],$$

where $\gamma_D[W^*]$ is the conjugation rate for the largest number of wells (W^*), and $\gamma_D[W'_i]$ is the conjugation rate for the ith assay using a smaller number of wells (W'). Thus, we see that as we partition wells into smaller numbers per estimate, the mean estimate will rise, which is what we see in SI Figure 11d. As a consequence, we recommend a reasonably large number of wells in the LDM assay. A number between 50 and 100 appears sufficient to avoid inaccuracy and is also convenient when using a microtiter plate format for populations.

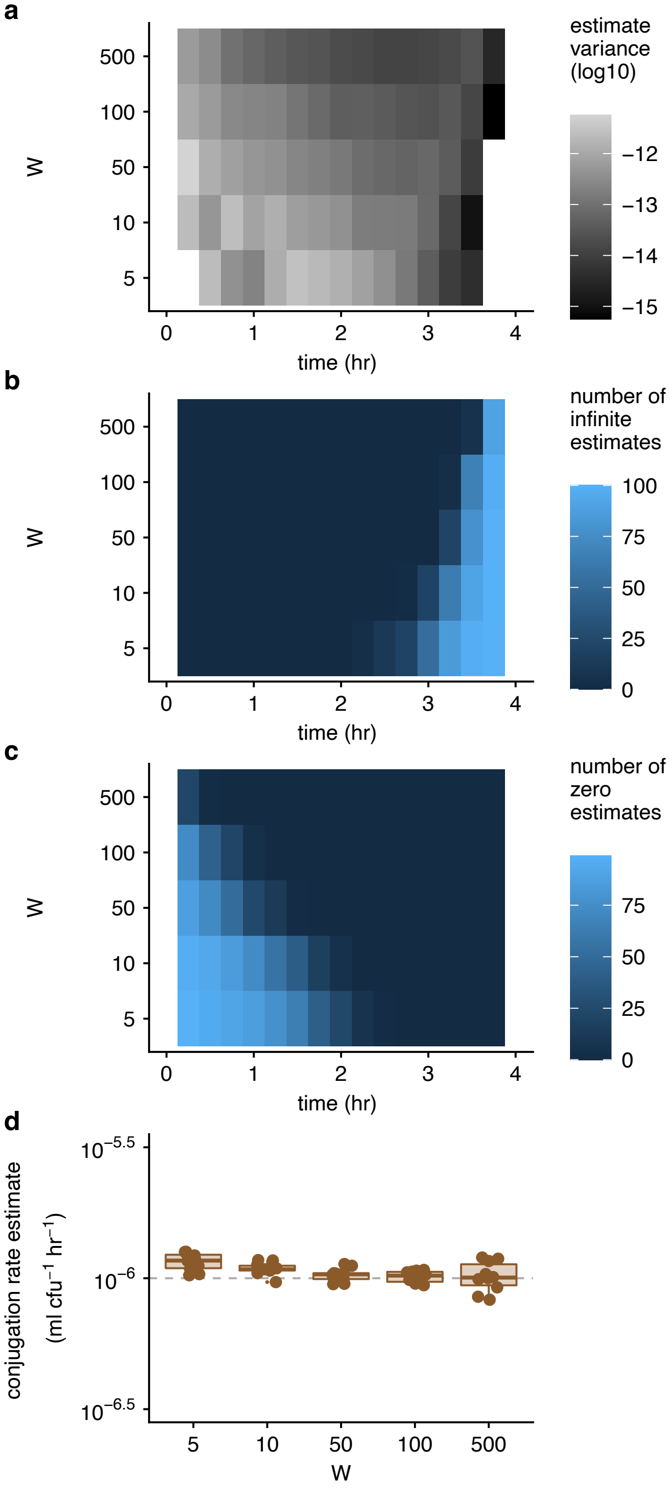

SI Figure 11: The variance of LDM estimates using stochastic simulation. Different number of populations (W) are used for the LDM estimates, as indicated. The parameters used here are the same baseline parameters in SI Figure 1 which were $\psi_D = \psi_R = \psi_T = 1$, and $\gamma_D = \gamma_T = 10^{-6}$. The dynamic variables were initialized with $D_0 = R_0 = 10^2$ and $T_0 = 0$. (a) The variation of each estimate is given at 15-minute intervals where more than 1 out of the 100 calculated estimates produced a finite non-zero value.

We ignore infinite estimates in the calculation of the variance. (b) The number of estimates with an infinite value out of the 100 calculated. (c) The number of estimates with a zero value out of the 100 calculated. (d) A total of 500 populations is partitioned in different ways—split into 100 groups of 5 populations (W=5), 50 groups of 10 populations (W=10), 10 groups of 50 populations (W=50), 5 groups of 100 populations (W=100), or a single group of 500 populations (W=500). Each plotted point is the mean conjugation rate of the rates calculated for each group (where the number of populations within each group vary as indicated by the W value). Each partitioning was run 10 times.

2. Following: in a traditional conjugation assay (e.g. SIM), one would normally establish multiple replicate conjugation reactions for each treatment, enumerate the various (sub-)populations within each replicate, and calculate conjugation rate from each replicate. The mean and variance of these calculated values would then be statistically analysed and compared between conditions e.g. by a linear model. A similar approach appears to have been used for the LDM in Figure 6 to compare between these approaches — 6 replicates were run (lines 616-617). But perhaps, if one was to exclusively use the LDM method to compare treatments, it would be possible to test statistical significance on a basis analogous to a binomial test? That is, rather than running 84 wells six times (n = 6), a measure of variance could be calculated from the proportion of positives across all 504 wells. I'm not sure exactly how this would work in the context of the full formula, nor whether it would increase statistical power... I accept that addressing this may well be beyond the scope of the current manuscript but I am curious to know the authors' thoughts.

We thank the reviewer for the interesting question. First, there is an important caveat. The LDM estimate requires more quantities than $\hat{p}_0(\tilde{t})$, i.e., namely donor and recipient densities over time as well as the incubation time of the assay. If these other quantities change from one treatment to another, than it won't be sufficient to compare only the number of positive wells (because $\hat{p}_0(\tilde{t})$ will not be the only factor leading to a change in γ_D). If conjugation rate and/or environmental conditions are changing with treatment, then these quantities (e.g., final donor and recipient densities as well as time of the assay) may be expected to vary (perhaps substantially). However, let's assume that all of these other quantities have the same expected values across treatments. In such a case, the conjugation rates in the different treatments would be fairly close to one another. In such a case, it does seem reasonable to us that a statistical test grounded in the number of positive wells (e.g., binomial test or Fisher's exact test) should give a result consistent with the approach that we use, and, in such a case, it would be easier to simply pool wells. Would this improve statistical power? While we are not sure of the answer, we did think that it was worthwhile to ask what the effect of pooling wells on the precision of the estimate would be. This question motivated the simulations behind SI Figure 11d (in the response to the reviewer's point #1). For instance, we can compare a single estimate with 500 wells to the mean of 5 estimates with 100 wells each. Interestingly, we see that there is actually less variance to the mean of 5 estimates with 100 wells each. We believe this occurs because taking a mean also mutes the effects of variation in the other quantities (i.e., donor and recipient densities), which tends to improve the precision. This does mean that if a simple comparison of positive wells is not possible (e.g., because other quantities in the estimate are changing across treatments), that there may be reasons not to pool wells for a minimal number of estimates. However, one should also not split the wells so much that each estimate has very few wells (as bias can start to enter the mean estimate). From our preliminary simulations, it seems that running a set of replicates with about 50-100 wells each may be the best strategy.

3. The authors comprehensively and clearly address the potential issues in their approach arising from transconjugant-selecting media and extinction probability in the SI (SI section 6d). However, this was not well signposted in the main text. Perhaps a couple of words could be inserted line 615: "When designing transconjugant-selecting media, appropriate preliminary and control experiments must be conducted to ensure that the media enables exclusive growth of transconjugants (see SI section 6d)".

We thank the reviewer for bringing up this point. We have added a few relevant additions to the main text. First, we have added a reference to the extinction probability section as we discuss some of the connections

between the Luria-Delbrück mutation model and our conjugation model (namely that both models are stochastic birth process models, but a failure of transconjugant establishment is an important kind of effective "cell death" which we deal with in the SI). The end of the first Results section now reads (where the relevant parts are highlighted (lines 251 – 272):

"Comparing equation [12] to equation [11], conjugation can be thought of as a mutation process with initial wild-type population size $D_0 R_0$ that grows at rate $\psi_D + \psi_R$. The structural similarity of the estimates is grounded in a structural similarity of the underlying models; indeed, some of the same assumptions that apply to the mutation process modeled by Luria and Delbrück also apply to the conjugation process modeled here. For instance, the loss of recipients due to plasmid transfer is ignored in the recipient dynamics of the conjugation model (equation [6]) in the same way that the loss of wildtype cells due to mutation is ignored in the wildtype cell dynamics of the mutation model (equation [3.1] in SI Section 3), which tends to be a safe assumption when growth greatly outpaces transformation. Furthermore, in the same way that reversions (mutations restoring a wildtype genotype from a mutant) are disregarded in the mutational model, we ignore the possibility of transconjugants (and donors) becoming plasmid-free through segregational loss in the conjugation model. Lastly, as in the original Luria-Delbrück model, we focus on a pure "birth" process (e.g., once mutants or transconjugants are generated, their numbers do not decrease). In our supplemental sections we explore the impacts of violations to some of these assumptions (e.g., the negligible impact of segregational loss in SI Section 4d, and how to correct for an effective loss in transconjugant cell numbers due to a failure of small numbers of transconjugants to establish under experimental conditions in SI Sections 6d and 7). Given the connections between modeling frameworks and estimate structures, we label the expression in equation [11] as the LDM estimate for donor conjugation rate, where LDM stands for "Luria-Delbrück Method."

Additionally, as the reviewer suggests, we also add the following to the Methods (lines 652 – 656):

"This medium disrupted new conjugation events—immediately by diluting cells then by inhibiting donors and recipients—while simultaneously allowing growth for transconjugants. When designing transconjugant-selecting media, appropriate preliminary and control experiments must be conducted to ensure that the media enables exclusive growth of transconjugants (see SI section 6c)."

Here we refer to SI section 6c (focused on preventing donor and recipient growth and allowing transconjugant growth) as opposed to SI section 6d (focused on the extinction probability assay and estimate correction). However, we now do refer to the extinction probability SI sections (6d and 7) at a few points in the main text.

Reviewer 3:

By providing an improved method of estimating conjugation rates (as well as carefully comparing it to previous methods), this study fills an important gap in the field. It is also explained clearly and with enough detail, such that others could apply the method themselves. Kudos to the authors for a very interesting, thorough and rigorous study. They've done a great job of keeping the main text concise and focused on the key messages, but the Supplementary contains a wealth of useful additional information – I appreciate the amount of work that goes into this material, and I think it really contributes to good science. The manuscript is already very clearly written and the figures are well designed to get across some rather complex ideas – clearly a lot of thought has gone into presentation.

Scientifically, I don't see any faults with this study – the math all looks correct, and the experimental protocol is thoroughly described and includes carefully chosen controls.

We sincerely thank the reviewer for their positive comments.

On close reading of both main + SI, I've still ended up with a long list of minor suggestions to further improve the presentation or clarify occasional details, but I emphasise that this is pretty high-level polishing and I am happy to leave most of these suggestions to the authors' discretion (other than fixing an apparent broken link in the GitHub repository).

1. Abstract – I personally thought the importance of using a stochastic approach could have been better emphasised – the current framing suggests the main advance is to address asymmetries in rates, with stochasticity an afterthought rather than a crucial factor in achieving accuracy. I had a similar impression in the Introduction – it wasn't clearly stated why a stochastic description is important in general, not only for unequal rates. For example, what if gT = gD but both are low? (However, if the authors consider the emphasis on heterogeneous rates to be more relevant to the field, then I accept their judgement on this point.)

We thank the reviewer for their candor about a missed opportunity for emphasis. We have rewritten the abstract, significance statement and one paragraph in the introduction to highlight how the stochastic approach differs from prior deterministic ones. The abstract and significance statement now read as follows (with the relevant portions in highlighted (lines 32 – 66):

"**Abstract**

To increase our basic understanding of the ecology and evolution of conjugative plasmids, we need a reliable estimate of their rate of transfer between bacterial cells. Current assays to measure transfer rate are based on deterministic modeling frameworks. However, some cell numbers in these assays can be very small, making estimates that rely on these numbers prone to noise. Here we take a different approach to estimate plasmid transfer rate, which explicitly embraces this noise. Inspired by the classic fluctuation analysis of Luria and Delbrück, our method is grounded in a stochastic modeling framework. In addition to capturing the random nature of plasmid conjugation, our new methodology, the Luria-Delbrück method ('LDM'), can be used on a diverse set of bacterial systems, including cases for which current approaches are inaccurate. A notable example involves plasmid transfer between different strains or species where the rate that one type of cell donates the plasmid is not equal to the rate at which the other cell type donates. Asymmetry in these rates has the potential to bias or constrain current transfer estimates, thereby limiting our capabilities for estimating transfer in microbial communities. In contrast, the LDM overcomes obstacles of traditional methods by avoiding restrictive assumptions about growth and transfer rates for each population within the assay. Using stochastic simulations and experiments, we show that the LDM has high accuracy and precision for estimation of transfer rates compared to the most widely used methods, which can produce estimates that differ from the LDM estimate by orders of magnitude.

Significance Statement

Conjugative plasmids play significant roles in the dynamics of microbial communities. Conjugation, the horizontal transfer of plasmids from one cell to another, is a common means of spread for genes of ecological significance, including genes encoding antibiotic resistance. For both public health modeling and a basic understanding of microbial population biology, accurate estimates of the rate of plasmid transfer are of great consequence. Widely used methods assume the process of conjugation is deterministic and, under certain conditions, lead to biased estimates that deviate from true values by several orders of magnitude. Therefore, we developed a new approach, inspired by the classic fluctuation analysis of Luria and Delbrück, which treats plasmid transfer as a random process. Our Luria-Delbrück method is straightforward to implement in the laboratory and can accurately estimate the rate of plasmid conjugation for different bacterial systems under a wide variety of conditions."

We have also incorporated some of this emphasis into the end of the introduction. Here we provide the first part of this restructured last paragraph (lines 144 – 152):

"Here we derive a novel estimate for conjugation rate by explicitly tracking transconjugant dynamics as a stochastic process (i.e., a continuous time branching process). This represents a notable deviation from previous approaches that are built upon deterministic frameworks. The random nature of conjugation can lead to substantial variation in the number of transconjugants at the end of a mating assay ($T_{\tilde{t}}$) as this population will often be small. Prior deterministic frameworks rely on this number (e.g., equation [4]), such that transconjugant variation adds problematic noise to the estimate. In contrast, our stochastic approach leverages this noise to produce an estimate (akin to the way Luria and Delbrück estimated mutation rate (4))…."

2. L45-6: the wording "not being affected by different growth and transfer rates" could be confusing; I think you mean the *method* is equally applicable, not that the *results* are unaffected.

Thank you for pointing this out-- we agree that this should be made clearer. Thus, we have made the following highlighted word changes to the relevant sentence (lines 47 – 49):

"In contrast, the LDM overcomes obstacles of traditional methods by avoiding restrictive assumptions about growth and transfer rates for each population within the assay."

3. Eq. [4]: should the LHS just be g, not gD?

The reviewer is correct that the original version uses γ and not γ_D. When $\gamma_D = \gamma_T = \gamma$, which is an assumption being made in the model underlying the SIM approach, either quantity could be used in principle. But what quantity is the actual target of the SIM estimate? This estimate is meant to describe the rate of conjugation from donors to recipients, which is why we write this as γ_D.

When $\gamma_D \ll \gamma_T$, then there are two ways to describe the set-up of this equation for the SIM estimate:

1. The SIM approach is still trying to estimate the value of γ_D, but it is inaccurate (as one of the assumptions of its underlying model is violated). Here one might prefer to use γ_D for the equation to underline that this is the target quantity.
2. The SIM approach actually is capturing some hybrid quantity that blend effects of the different conjugation rates from donors (γ_D) and transconjugants (γ_T). This hybrid quantity is termed γ, and its deviation from the true value of γ_D is the indication that the violation of the model assumption has caused a deviation from the intended target.

We think a case can be made for each description, but we lean towards the first so that all estimates underline their common target quantity explicitly.

4. L226, wording could be slightly clearer: "where there are *initially* no transconjugants"; "the only process that can *initially* change the number of transconjugants" (or, "produce the first transconjugant")

We thank the reviewer for these changes, which definitely improves the clarity of this passage. We have added the suggested highlighted edits to the relevant sentences (lines 236 – 238):

"Given the standard set-up of a mating assay, we focus on a situation where there are initially no transconjugants. Therefore, the only process that can produce the first transconjugant is conjugation of the plasmid from a donor to a recipient."

5. A critical assumption in the derivation of the p0 estimation method (similarly to its use for mutation rate estimation) is that the mutant or transconjugant population, once becoming non- zero, never drops to zero again. In the present case, there are (at least) two key assumptions for this to be true: (1) no cell death; (2) no segregative loss of the plasmid. #1 should be reasonable in benign growth conditions but could be given a passing mention. #2 seems important, and was actually addressed in the Supplementary – I think this is well worth mentioning in the main text.

We thank the reviewer for raising this important point. We now bring up these issues (in the context of the connection of our modeling assumptions to the modeling assumptions of Luria and Delbrück). We now explicitly note that our model (like the Luria-Delbrück model) is a pure stochastic birth process, which means that loss of transconjugants (e.g., through cell death or segregational loss) is ignored. We added some text to the end of the first section in the Results, which reads as follows (where we have highlighted the most relevant parts) (lines 251 – 272):

"Comparing equation [12] to equation [11], conjugation can be thought of as a mutation process with initial wild-type population size $D_0 R_0$ that grows at rate $\psi_D + \psi_R$. The structural similarity of the estimates is grounded in a structural similarity of the underlying models; indeed, some of the same assumptions that apply to the mutation process modeled by Luria and Delbrück also apply to the conjugation process modeled here. For instance, the loss of recipients due to plasmid transfer is ignored in the recipient dynamics of the conjugation model (equation [6]) in the same way that the loss of wildtype cells due to mutation is ignored in the wildtype cell dynamics of the mutation model (equation [3.1] in SI Section 3), which tends to be a safe assumption when growth greatly outpaces transformation. Furthermore, in the same way that reversions (mutations restoring a wildtype genotype from a mutant) are disregarded in the mutational model, we ignore the possibility of transconjugants (and donors) becoming plasmid-free through segregational loss in the conjugation model. Lastly, as in the original Luria-Delbrück model, we focus on a pure "birth" process (e.g., once mutants or transconjugants are generated, their numbers do not decrease). In our supplemental sections we explore the impacts of violations to some of these assumptions (e.g., the negligible impact of segregational loss in SI Section 4d, and how to correct for an effective loss in transconjugant cell numbers due to a failure of small numbers of transconjugants to establish under experimental conditions in SI Sections 6d and 7). Given the connections between modeling frameworks and estimate structures, we label the expression in equation [11] as the LDM estimate for donor conjugation rate, where LDM stands for "Luria-Delbrück Method."

6. L248-9: Using the Gillespie algorithm, presumably you have treated all populations (D, R, T) stochastically? This would be helpful to state explicitly, as it contrasts with the analytical treatment above, where only T is stochastic.

We thank the reviewer for this clarifying suggestion. We have added the suggested highlighted edits to the relevant sentences (lines 275 – 278):

"To explore the accuracy and precision of the LDM estimate and compare it to the SIM estimate (as well as other estimates, see SI section 4), we used the Gillespie algorithm to stochastically simulate the dynamics of donors, recipients, and transconjugants in a standard mating assay using equations [1]-[3]."

7. L266-7: The LDM estimate really requires a mix of presence/absence across replicates.

We appreciate the reviewer pointing out this correction. We have edited the highlighted area in the relevant sentence (lines 296 – 299):

"Because the LDM estimate requires the *absence* of transconjugants in a fraction of populations, while the SIM estimate requires their *presence*, the range of incubation times for the LDM approach will be earlier than the SIM approach."

8. L289 word choice: "surpassed" might be confusing (suggesting the estimate was higher); rather, "outperformed"?

We thank the reviewer for the correction. We have edited the highlighted area in the relevant sentence (lines 319 – 320):

"Once again, the LDM estimate generally outperformed the SIM estimate across this range (Figure 4b)."

9. L304: "at a time close to *the average* t*"

We appreciate the reviewer pointing out this mistake. We have added highlighted words to the relevant sentence (lines 334 – 337):

"The basic approach is to inoculate many donor-recipient co-cultures and then, at a time close to the average t^*, add transconjugant-selecting medium (counterselection for donors and recipients) to determine the presence or absence of transconjugant cells in each co-culture."

10. Around L339: Is there any risk that wells were already grown enough to be (somewhat) turbid before addition of the selective media? If so, will turbidity decline as cells die, or could dead cells or debris continue to contribute enough OD to interfere with scoring turbidity at the end of the assay?

We appreciate the reviewer asking this question given that a turbid vs. non-turbid signal after growth in transconjugant-selecting medium is necessary for calculating $\hat{p}_0(\tilde{t})$. For the range of conjugation rate estimates we measured in the laboratory (i.e., ~1 x 10^{-14} – 1 x 10^{-7}) and reported in this study, the 1:10 dilution in the deep-well plates was sufficient to dilute cultures such that this kind of spurious turbidity (which could have interfered in scoring the assay) did not occur. For instance, with our approach, we never observed turbidity (comparable to the wells with transconjugant growth) in the control wells (e.g., wells of donor alone or recipient alone) after the transconjugant-selecting medium was added, nor at the end of the protocol. More generally, we provide guidance on how to check and troubleshoot the transconjugant-selecting medium in the step-by-step protocol provided on protocols.io. In response to this question, we have also added more details in the relevant protocol steps (steps 16 – 17) about how to change the dilution factor and microtiter plate format if 'background turbidity' is an issue for a future user.

"Here we are recommending that the user dilute the mating cultures ten-fold with transconjugant-selecting medium. After overnight incubation, if the turbidity of the transconjugant control well is too similar to the mating control wells making it difficult to distinguish turbidity coming from transconjugant growth and turbidity coming from inhibited/dead donor and recipient cells, then this may be indicative that the final densities of the donors and recipients are too high relative to the dilution factor. One option for mitigating this issue is to dilute the mating cultures more than ten-fold. This would require a larger deep-well format or a smaller mating culture volume."

11. As I was reading the main text, a couple of questions about the lab protocol came to mind: (1) Are both estimation methods (SIM and LDM) applied to the exact same cultures, or separate ones?

We agree that this could be made clearer. Thus, we have made the following highlighted word changes to a relevant sentence in the first paragraph in the 'Cross-species case study' section (lines 285 – 287):

"We implemented the LDM and SIM protocols on the same bacterial cultures to compare the laboratory estimates of cross-species conjugation rate."

> (2) Were the growth rates (y's) estimated separately? These points became clear later in the more detailed methods, but might be worth a brief mention when they first come up.

We concur that an earlier mention would be better. Thus, we have referred the reader to the appropriate SI section earlier by adding the following highlighted words to the third paragraph in the 'Cross-species case study' section (lines 409 – 411):

"Second, our growth rate assays (conducted separately from the transfer estimate protocols; see SI section 6b) revealed our cell types have different growth rates (SI Figure 8), thus violating the SIM assumptions."

> Also, while I appreciate that \tilde{t} is chosen differently for each parameter set, I would find it helpful to have some idea of the range in the main text (at least in Materials & Methods, around L604) and perhaps in relevant figure captions.

This is a very good suggestion, and a similar request was made by reviewer 1. To provide comprehensive information about the different incubation times used in each scenario, we have made the following highlighted word changes in the main text and provided a supplementary table:

Lines 711 – 713:
"To compare across various parameter settings (Figure 4), a single incubation time was chosen per parameter set and type of estimate (See SI Table 5 for the incubation times used)."

SI Table 5: Specific incubation times ($\tilde{t}$) used in stochastic simulations to compare across parameter settings. Each row lists the relevant figure and the corresponding x-axis value. Time is given in hours. For each parameter setting, the incubation time $\tilde{t}$ for the LDM estimate is set to the average t^*, and for the SIM estimate is given by the time point for which an average of 50 transconjugants is reached.

**Figure**	**x-axis value**	$\tilde{t}_{LDM}$	$\tilde{t}_{SIM}$
Figure 4, SI Figure 1a	0.0625	4.34	7.89
Figure 4, SI Figure 1a	0.125	4.11	7.49
Figure 4, SI Figure 1a	0.25	3.7	6.78
Figure 4, SI Figure 1a	0.5	3.1	5.67
Figure 4, SI Figure 1a	1	2.35	4.27
Figure 4, SI Figure 1a	2	1.61	2.86
Figure 4, SI Figure 1a	4	1.01	1.74
Figure 4, SI Figure 1a	8	0.6	1
Figure 4, SI Figure 3	1×10^9	2.35	4.27
Figure 4, SI Figure 3	1×10^8	2.35	4.27
Figure 4, SI Figure 3	1×10^7	2.35	4.27
Figure 4, SI Figure 3	1×10^6	2.35	4.27
Figure 4, SI Figure 3	1×10^5	2.35	4.25
Figure 4, SI Figure 3	1×10^4	2.33	4.11

Figure 4, SI Figure 3	1 x 10^3	2.16	3.4
Figure 4, SI Figure 3	1 x 10^2	1.44	2.02
SI Figure 1b	0.0625	4.35	8.18
SI Figure 1b	0.125	4.11	7.66
SI Figure 1b	0.25	3.71	6.85
SI Figure 1b	0.5	3.1	5.69
SI Figure 1b	1	2.35	4.27
SI Figure 1b	2	1.61	2.86
SI Figure 1b	4	1.01	1.74
SI Figure 1b	8	0.6	0.99
SI Figure 2a	0.0625	3.3	6.4
SI Figure 2a	0.125	3.22	6.23
SI Figure 2a	0.25	3.07	5.89
SI Figure 2a	0.5	2.8	5.26
SI Figure 2a	1	2.35	4.27
SI Figure 2a	2	1.78	3.07
SI Figure 2a	4	1.2	1.99
SI Figure 2a	8	0.74	1.15
SI Figure 2b	0.0625	3.31	6.45
SI Figure 2b	0.125	3.23	6.27
SI Figure 2b	0.25	3.07	5.92
SI Figure 2b	0.5	2.8	5.27
SI Figure 2b	1	2.35	4.27
SI Figure 2b	2	1.78	3.07
SI Figure 2b	4	1.2	1.97
SI Figure 2b	8	0.74	1.15
SI Figure 2c	0.0625	2.64	4.59
SI Figure 2c	0.125	2.62	4.57
SI Figure 2c	0.25	2.59	4.54
SI Figure 2c	0.5	2.52	4.46
SI Figure 2c	1	2.35	4.27
SI Figure 2c	2	1.97	3.62
SI Figure 2c	4	1.34	2.31
SI Figure 2c	8	0.8	1.29
SI Figure 4	0.00001	2.35	4.27
SI Figure 4	0.0001	2.35	4.27
SI Figure 4	0.001	2.35	4.27
SI Figure 4	0.01	2.36	4.29
SI Figure 4	0.1	2.47	4.49

12. L417: "the Levin *et al.* model" needs a reference number. This is also the first time this model is mentioned, and its key feature(s) or relation to the SIM could be briefly explained.

We thank the reviewer for the suggestion. We have added a reference number and have added a sentence to underline that the model behind the SIM approach makes an identical assumption of parametric homogeneity. This passage now reads as follows (lines 450 – 460):

"The most widely used approaches to estimate conjugation rate are derived from the Levin *et al.* model (5) (discussed in SI section 1) which assumes that all strains grow and conjugate at the same rate ($\psi_D = \psi_R = \psi_T$ and $\gamma_D = \gamma_T$). For instance, the model underlying the SIM approach assumes precisely this kind of homogeneity."

13. Around L460: SI section 8 analytically considers variance in the LDM to the ASM, whereas Fig. 4 compares LDM to SIM. Do the analytical insights transfer? Is SIM a special case of ASM if there is no resource depletion?

We thank the reviewer for this interesting question. One can derive the variance for the SIM estimate in a way analogous to the ASM estimate, with the caveat that an approximation is needed (namely one that is similar to the approximation used for the variance of the LDM estimate). We will assume that the SIM estimate is obtained during exponential growth of all populations (which are assumed to grow at the same rate), which will allow us to connect the variance for the SIM to the variance for the ASM. We provide some of the details here.

If we focus solely on the contribution of transconjugant variation to estimate variance, we can represent the SIM estimate as a random variable Γ_{SIM}:

$$\Gamma_{\mathrm{SIM}} = \mathrm{A}\ln(1 + \mathrm{B}T_{\tilde{t}})$$

where the coefficients are treated as the following constants:

$$\mathrm{A} = \frac{\psi}{N_0(e^{\psi\tilde{t}} - 1)},$$

$$\mathrm{B} = \frac{N_0}{D_0 R_0 e^{\psi\tilde{t}}}.$$

The variance of the estimate is then

$$\mathrm{var}(\Gamma_{\mathrm{SIM}}) = \mathrm{A}^2\mathrm{var}[\ln(1 + \mathrm{B}T_{\tilde{t}})]$$

Using the first-order Taylor expansion centered at $E[T_{\tilde{t}}]$:

$$\ln(1 + \mathrm{B}T_{\tilde{t}}) \approx \ln(1 + \mathrm{B}E[T_{\tilde{t}}]) + \frac{B}{1 + \mathrm{B}E[T_{\tilde{t}}]}(T_{\tilde{t}} - E[T_{\tilde{t}}]).$$

And we have

$$\mathrm{var}[\ln(1 + \mathrm{B}T_{\tilde{t}})] \approx \left(\frac{\mathrm{B}}{1 + \mathrm{B}E[T_{\tilde{t}}]}\right)^2 \mathrm{var}(T_{\tilde{t}}).$$

Thus, we have

$$\mathrm{var}(\Gamma_{\mathrm{SIM}}) \approx \left(\frac{\mathrm{AB}}{1+\mathrm{B}E[T_{\tilde{t}}]}\right)^2 \mathrm{var}(T_{\tilde{t}})$$

The quantity $E[T_{\tilde{t}}]$ is given in SI Section 7 (here we assume $\psi_D = \psi_R = \psi_T = \psi$). Plugging in the expressions for A, B, and $E[T_{\tilde{t}}]$ and simplifying yields,

$$\mathrm{var}(\Gamma_{\mathrm{SIM}}) \approx \frac{1}{\left(1+\frac{\gamma_D(N_{\tilde{t}}-N_0)}{\psi}\right)^2}\left(\frac{\psi}{D_0R_0\left(e^{2\psi\tilde{t}}-e^{\psi\tilde{t}}\right)}\right)^2 \mathrm{var}(T_{\tilde{t}})$$

The expression for variance of the ASM estimate (derived in SI Section 8) is:

$$\mathrm{var}(\Gamma_{\mathrm{ASM}}) = \left(\frac{\psi_D+\psi_R-\psi_T}{D_0R_0\left(e^{(\psi_D+\psi_R)\tilde{t}}-e^{\psi_T\tilde{t}}\right)}\right)^2 \mathrm{var}(T_{\tilde{t}})$$

If $\psi_D = \psi_R = \psi_T = \psi$ (as is assumed for the SIM estimate) we have

$$\mathrm{var}(\Gamma_{\mathrm{ASM}}) = \left(\frac{\psi}{D_0R_0\left(e^{2\psi\tilde{t}}-e^{\psi\tilde{t}}\right)}\right)^2 \mathrm{var}(T_{\tilde{t}})$$

Therefore,

$$\mathrm{var}(\Gamma_{\mathrm{SIM}}) \approx \frac{1}{\left(1+\frac{\gamma_D(N_{\tilde{t}}-N_0)}{\psi}\right)^2} \mathrm{var}(\Gamma_{\mathrm{ASM}})$$

At the time of the end of the assay ($t = \tilde{t}$), the product of donors and recipients ($D_{\tilde{t}}R_{\tilde{t}}$) is in the vicinity of the reciprocal of the conjugation rate ($1/\gamma_D$), but the sum of donors and recipients is much smaller than the reciprocal of the conjugation rate ($D_{\tilde{t}} + R_{\tilde{t}} \ll 1/\gamma_D$). We note $N_t \approx D_t + R_t$. Therefore, for reasonable times in which the assay is ended and a reasonable growth rate:

$$N_{\tilde{t}} - N_0 \ll \frac{\psi}{\gamma_D}$$

In such a case, $1+\frac{\gamma_D(N_{\tilde{t}}-N_0)}{\psi} \approx 1$, and

$$\mathrm{var}(\Gamma_{\mathrm{SIM}}) \approx \mathrm{var}(\Gamma_{\mathrm{ASM}})$$

Indeed, for an example close to that explored in SI Figure 10 (with $\psi_D = \psi_R = \psi_T = 1$, $D_0 = R_0 = 10^4$, and $\gamma_D = 10^{-12}$), the variances for the SIM and ASM estimates are virtually indistinguishable (in the figure below the variance for the ASM estimate is the thick green curve and the variance for the SIM estimate is the thin yellow curve—the two curves coincide perfectly).

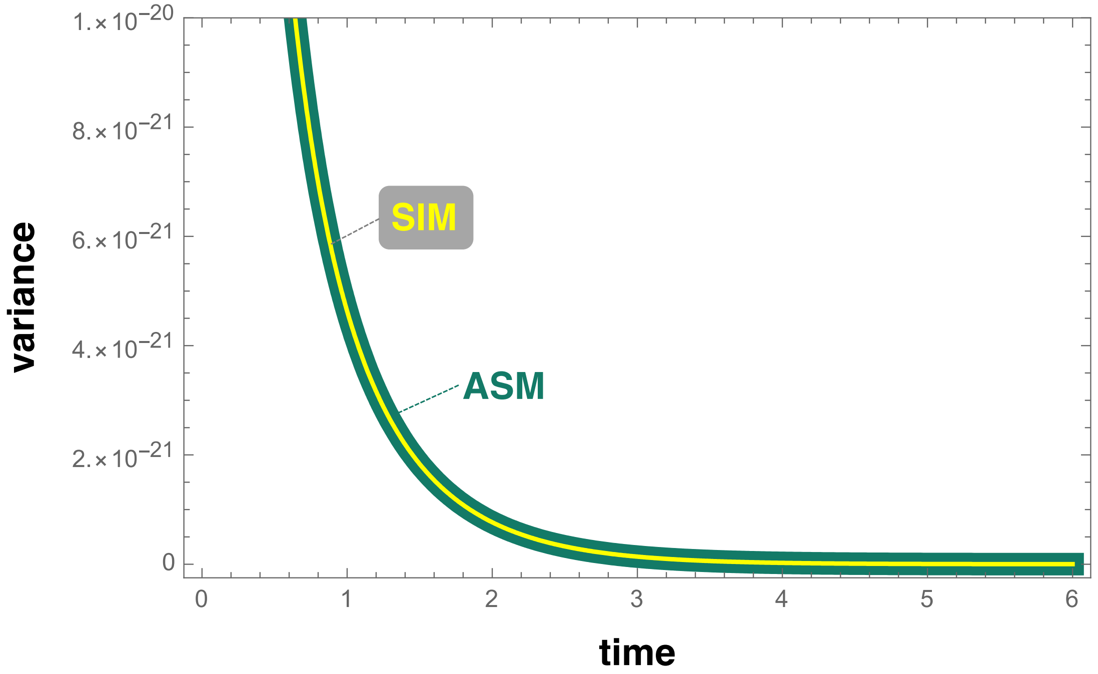

14. L614 minor semantic point: The medium inhibits donors & recipients (absolute growth rate < 0) while *allowing growth* of transconjugants (absolute growth rate > 0). Selection arises as a result of this combination – but selection (due to a *relative* fitness advantage) is not synonymous with growth (in absolute numbers); it's also possible to have a selective advantage while declining in absolute terms. (Similar issue in SI, L684: selection for resistance should not be equated with concentrations below the resistant strain's MIC.)

We agree that the 'i.e.,' statement we used before was sloppy and made an incorrect implication. We have highlighted the relevant portions of the changed sentences.

Lines 652 – 654 in the main text:
"This medium disrupted new conjugation events—immediately by diluting cells then by inhibiting donors and recipients—while simultaneously allowing growth of transconjugants."

SI lines 691 – 694 in the supplement:
"Given the low numbers of transconjugants in the co-cultures, the results from a recent study of Alexander and MacLean (6) have high relevance. First, the authors show that levels of antibiotic below the MIC of the resistant strain are sufficient to decrease the chance of outgrowth with very low cell numbers (e.g., a single cell)."

15. L623: Are colony counts from each of these three wells pooled or averaged to get a single estimate of population density?

We thank the reviewer for pointing out that this piece of information was missing. We made the following highlighted word changes:

Lines 642 – 645:
"First, 30 µl was removed from each of the wells used to determine initial densities, to uncover the final densities ($D_{\tilde{t}}$ and $R_{\tilde{t}}$) via selective plating (Figure 5b). We note that densities were calculated from each

well then averaged for calculating the LDM estimate. Second, donor and recipient monocultures were mixed at equal volumes into the two empty wells (Figure 5b, gray arrows)."

Lines 664 – 669:
'To derive the SIM estimate for each incubation group, 30 µl was removed from each of the three wells in the group at the time point ($\tilde{t} = 5$ and $\tilde{t} = 24$) to determine the final donor ($D_{\tilde{t}}$), recipient ($R_{\tilde{t}}$), and transconjugant ($T_{\tilde{t}}$) densities via selective plating. Densities were calculated from each well then averaged for calculating the SIM estimate. This protocol was repeated six times alongside the LDM replicates."

16. L658: Clarify (also in the Fig. 4 caption) that the SIM method only shows 100 estimates, not 10'000.

We thank the reviewer for pointing this out. For simplicity, we changed all the relevant figures (Figure 4, SI Figure 1-4) to show 100 estimates of each approach. We made the following highlighted word changes to the relevant sentences in each figure legend.

Lines 793 – 794 in figure 3 legend:
"In parts c and e, each box represents the distribution from 100 estimates of the donor conjugation rate for a given $\tilde{t}$, spanning from the 25th to 75th percentile."

Lines 801 – 803 in figure 4 legend:
"The Gillespie algorithm was used to simulate population dynamics. 100 estimates of the donor conjugation rate are shown for each parameter combination (summarized using boxplots with the same graphical convention as in Figure 3)."

17. L671: "a single incubation time" in what sense? It sounds like it differs for each parameter setting, and between LDM and SIM methods.

Yes, the reviewer is correct that each incubation time is specific to the parameter setting in the parameter sweep plots. As discussed under comment '11', additional text and an SI table were added to provide more information about incubation times.

18. GitHub repository: This is a great addition, but some of the links seem to be broken. If I click on Supporting Data or Simulations, I get only a garbled Readme.md file (see screenshot below). Please check/fix this before publication. (I'm on a Safari browser, if that makes any difference.)

We apologize for these issues with the GitHub documentation. We have remedied the ReadMe pages in the repository. In addition, the code for generating all the simulation data and the supporting documentation can be found at https://github.com/livkosterlitz/LDM/tree/main/Simulations. In addition, the supporting data folder was supposed to be deleted given that all the data used in the paper are given within the folders for each figure (see https://github.com/livkosterlitz/LDM/tree/main/Figures).

19. Fig. 3 caption, around L750 (and similarly for Fig. 4 caption): State how many estimates are derived from these 10'000 simulations.

We thank the reviewer for pointing this out. We made the following highlighted word changes to the relevant sentences in the figure legends.

Lines 793 – 794 for Figure 3:
"In parts c and e, each box represents the distribution from 100 estimates of the donor conjugation rate for a given $\tilde{t}$, spanning from the 25th to 75th percentile."

Lines 801 – 803 for Figure 4:
"Gillespie algorithm was used to simulate population dynamics. 100 estimates of the donor conjugation rate are shown for each parameter combination (summarized using boxplots with the same graphical convention as in Figure 3)."

20. Fig. 4 caption, L765: incubation times are specific to each parameter setting – and also differ between LDM and SIM?

We thank the reviewer for comments surrounding the confusion regarding chosen incubation times for analyzing the stochastic simulations. As discussed under comment '11' and '17', additional text and an SI table were added to provide more information about incubation times.

21. To play devil's advocate, the superior precision of the LDM method might be expected given that the estimate is derived from a much larger sample size of populations. For instance in Fig. 4, each LDM estimate is based on 100 simulated populations while each SIM estimate is based on only one. Similarly, in SI section 8, the variance in the ASM estimate is derived for a single measurement of Tt (I think) whereas the variance in the LDM estimate is derived for W wells (plotted in SI Fig. 10 for W=10 and W=100). Multiple replicates are clearly necessary for an estimate based on a stochastic process, but the authors might acknowledge that the improvement in precision comes at the cost of doing a larger experiment (or rebut my thinking if you disagree). To that end – the authors could also consider pointing out, when describing the experimental protocol, that the number of replicate wells used to derive one estimate in the LDM can be varied; 84 is convenient for the layout of a single microtitre plate, but there is no reason a user couldn't adjust this, either decrease (at the cost of lower precision) to run higher- throughput assays across multiple environments, or increase to improve precision.

We thank the reviewer for the comment. We agree that the precision of the LDM estimate is improved by virtue of using information from multiple populations, and that the more populations used (the higher the value of W), the lower the variance of the estimate. This was something that we were alluding to in the "The LDM approach has improved precision" section of the discussion. However, we have added a few words (highlighted) to make this even clearer (lines 499 – 504):

"For the number of parallel co-cultures in our protocol, the LDM estimate had smaller variance compared to other estimates, even under parameter settings where different estimates shared similar accuracy (e.g., Figure 4). This greater precision likely originates from the difference in the distribution of the number of transconjugants ($T_{\tilde{t}}$) and the distribution of the probability of transconjugant absence ($p_0(\tilde{t})$, where variance decreases with the number of co-cultures), something we explore analytically in SI section 8."

We also thought that the relationship between the number of co-cultures and the variance in the LDM estimate warranted further exploration (especially as a function of the time of the assay). We have enlarged the discussion and analysis in SI Section 8, where we use simulations to explore how LDM estimate variance changes with the number of populations followed. For the number of wells we are using (in the range between 50 and 100) the estimate appears to be accurate with low variance. We produce a new figure, where we show how variance changes with both the number of populations/wells and the assay time (which will affect the value of $p_0(\tilde{t})$). We include this added part of the SI section here for the convenience of the reviewer:

As illustrated in SI Figure 10, the variance in the LDM estimate changes with the number of populations (W). How does this number affect the variance in the LDM estimate? Here we use simulations to further explore this question. In SI Figure 11a, we present the variance of LDM estimates as a function of assay

time ($\tilde{t}$) and the number of populations (W). Generally, as the number of populations decreases or as the boundaries of the time interval are approached (where nearly none or all of the populations have transconjugants) the variance in the LDM estimate rises. The exception seems to be for times that are very long, but the low variance is likely a result of having many infinite estimates that are not included in the estimate variance (SI Figure 11b). Both infinite estimates (SI Fig. 11b) and zero estimates (SI Fig. 11c) are more likely as the number of populations decreases; in other words, the interval of assay times producing non-zero finite estimates increases with the number of populations. Generally, the greater the number of populations and the more intermediate the assay time (e.g., where approximately half of the populations have transconjugants), the lower the variance.

Suppose an experimenter is considering some number of wells (populations) and wants to decide how many estimates to produce. For instance, with 500 wells, the experimenter could decide to run a single LDM assay and obtain a single estimate (with $\mathrm{W} = 500$) or perhaps instead could run 5 assays (with $\mathrm{W} = 100$), 10 assays (with $\mathrm{W} = 50$), 50 assays (with $\mathrm{W} = 10$) or 100 assays (with $\mathrm{W} = 5$) for 5, 10, 50, and 100 estimates, respectively. Does it make a difference to the precision or accuracy to split or lump wells? Here we explore this question through simulation. How do we compare different partitions of wells? Let us consider some total number of wells, call this W^*, and consider some factor of W^*, which we will call W'; i.e., $\mathrm{W}^*/\mathrm{W}' = n$, where n is an integer. Here we will compare a single estimate with W^* wells with the mean of n estimates that each use W' wells. Thus, for SI Figure 11d, each point for $\mathrm{W} = 500$ is a single estimate, where each point for $\mathrm{W} = 5$, $\mathrm{W} = 10$, $\mathrm{W} = 50$, and $\mathrm{W} = 100$ is the mean of 100, 50, 10, and 5 estimates, respectively. With these comparisons in mind, we see two slight effects of different partitioning patterns. First, the variance is a bit higher for the single estimate coming from the largest number of wells. We attribute this shift to the fact that other quantities involved in the estimate (e.g., density of donors and recipients) are only being computed once for each point for $\mathrm{W} = 500$ in Fig. SI 11d, whereas these quantities are being computed multiple times for smaller W values, such that anomalous values would tend to get muted as the estimates were averaged. The second effect is a more notable one. We see that as the number of wells per estimate goes down, slight inaccuracies in the estimate start to occur. Why does this happen?

To answer this question, let us consider the LDM estimate:

$$\gamma_D = -\ln p_0(\tilde{t})\left(\frac{\psi_D + \psi_R}{D_0 R_0\left(e^{(\psi_D+\psi_R)\tilde{t}} - 1\right)}\right)$$

The main thing that will be affected by the number of populations is $p_0(\tilde{t})$. Specifically, as W decreases, the variance in the fraction of populations without transconjugants increases. Suppose that we have n LDM estimates under consideration, and for each one a value $\hat{p}_0(\tilde{t})$ is needed. Here we define:

$$\overline{\hat{p}_0(\tilde{t})} = \frac{\sum_{i=1}^{n} \hat{p}_{0,i}(\tilde{t})}{n},$$

where $\hat{p}_{0,i}(\tilde{t})$ is the fraction of populations without transconjugants for the ith estimate. Now, by Jensen's inequality, we have:

$$-\ln\left\{\frac{\sum_{i=1}^{n} \hat{p}_{0,i}(\tilde{t})}{n}\right\}\left(\frac{\psi_D + \psi_R}{D_0 R_0\left(e^{(\psi_D+\psi_R)\tilde{t}} - 1\right)}\right) < \frac{1}{n}\sum_{i=1}^{n} -\ln \hat{p}_{0,i}(\tilde{t})\left(\frac{\psi_D + \psi_R}{D_0 R_0\left(e^{(\psi_D+\psi_R)\tilde{t}} - 1\right)}\right)$$

$$-\ln \overline{\hat{p}_0(\tilde{t})}\left(\frac{\psi_D+\psi_R}{D_0 R_0\left(e^{(\psi_D+\psi_R)\tilde{t}}-1\right)}\right)<\frac{1}{n}\sum_{i=1}^{n}-\ln \hat{p}_{0,i}(\tilde{t})\left(\frac{\psi_D+\psi_R}{D_0 R_0\left(e^{(\psi_D+\psi_R)\tilde{t}}-1\right)}\right)$$

As W gets large, the value $\hat{p}_0(\tilde{t})$ is close to $\overline{\hat{p}_0(\tilde{t})}$ for smaller W values. Thus, using the terminology from above:

$$\gamma_D[\mathrm{W}^*]<\frac{1}{n}\sum_{i=1}^{n}\gamma_D[\mathrm{W'}_i],$$

where $\gamma_D[\mathrm{W}^*]$ is the conjugation rate for the largest number of wells (W^*), and $\gamma_D[\mathrm{W'}_i]$ is the conjugation rate for the ith assay using a smaller number of wells ($\mathrm{W'}$). Thus, we see that as we partition wells into smaller numbers per estimate, the mean estimate will rise, which is what we see in SI Figure 11d. As a consequence, we recommend a reasonably large number of wells in the LDM assay. A number between 50 and 100 appears sufficient to avoid inaccuracy and is also convenient when using a microtiter plate format for populations.

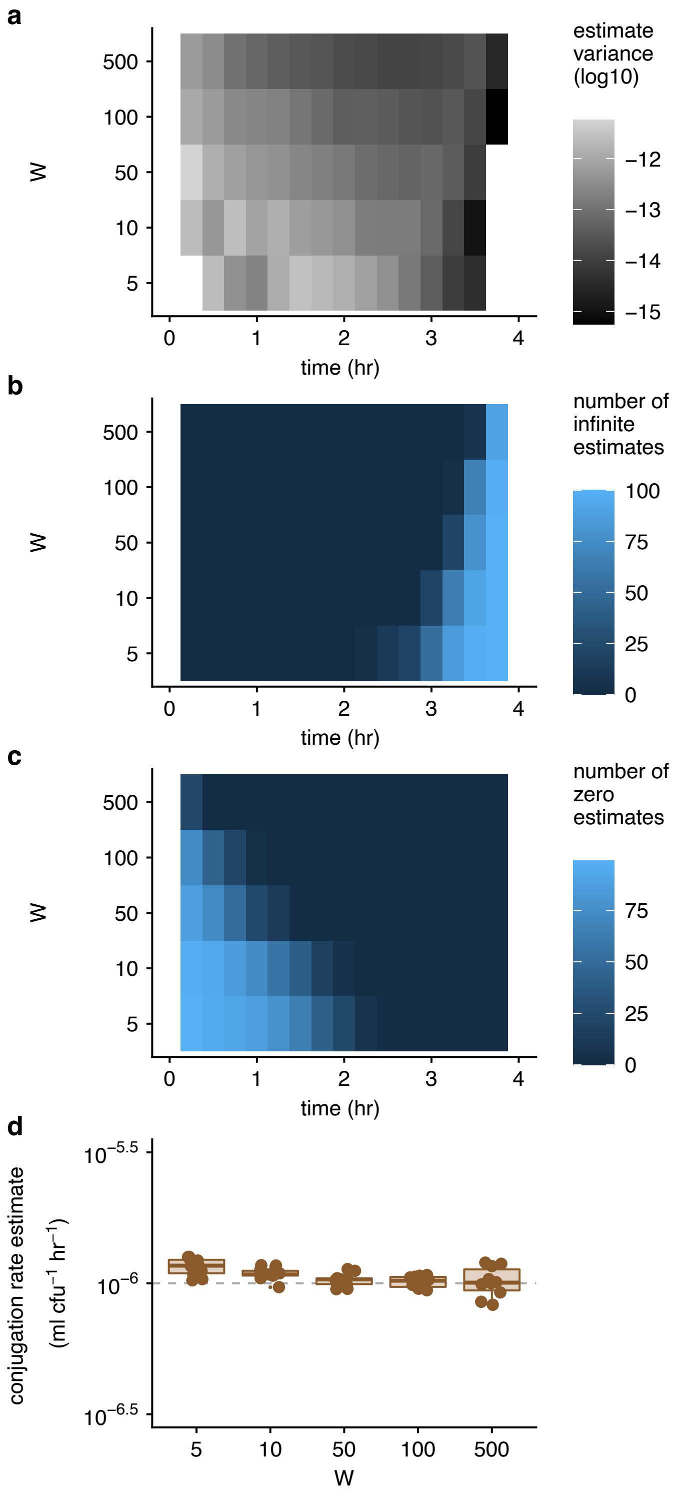

SI Figure 11: The variance of LDM estimates using stochastic simulation. Different number of populations (W) are used for the LDM estimates, as indicated. The parameters used here are the same baseline parameters in SI Figure 1 which were $\psi_D = \psi_R = \psi_T = 1$, and $\gamma_D = \gamma_T = 10^{-6}$. The dynamic variables were initialized with $D_0 = R_0 = 10^2$ and $T_0 = 0$. (a) The variation of each estimate is given at 15-minute intervals where more than 1 out of the 100 calculated estimates produced a finite non-zero value. We ignore infinite estimates in the calculation of the variance. (b) The number of estimates with an infinite

value out of the 100 calculated. (c) The number of estimates with a zero value out of the 100 calculated. (d) A total of 500 populations is partitioned in different ways—split into 100 groups of 5 populations (W=5), 50 groups of 10 populations (W=10), 10 groups of 50 populations (W=50), 5 groups of 100 populations (W=100), or a single group of 500 populations (W=500). Each plotted point is the mean conjugation rate of the rates calculated for each group (where the number of populations within each group vary as indicated by the W value). Each partitioning was run 10 times.

22. SI section 1a, L52-7: I'd find it helpful to state what these assumptions mean in terms of model parameters (i.e., y=0 and D, R constant?).

We strongly agree with the reviewer that assumptions are made clearer when stated mathematically, so we have added a number of parentheticals and an extra sentence to clarify this passage, which now reads as follows (SI lines 52 – 61):

"First, the change in cell density of donors due to growth is assumed to be negligible (i.e., $dD/dt \approx 0$). Likewise, the change in cell density of recipients due to growth and to conjugation (i.e., transformation into transconjugants) is assumed to be negligible (i.e., $dR/dt \approx 0$). Finally, transconjugants are assumed to be rare in the population such that the increase in transconjugant cell density is primarily through plasmid conjugation from donors to recipients (i.e., in equation [1.3], $\gamma DR \gg \psi T + \gamma TR$). All of these assumptions are satisfied if the cell growth rate is zero ($\psi = 0$), the conjugation rate (γ) is small, the system starts without transconjugants ($T_0 = 0$), and the densities of donors and recipients remain much greater than the density transconjugants for the period under consideration ($D \gg T$ and $R \gg T$)."

23. SI table 4, 5th row: "per replicate" is a confusing word choice here – perhaps, "to obtain one estimate"?

We agree with the reviewers suggested word change. We have made the following highlighted word changes to SI Table 4 (SI line 225).

SI Table 4: Comparison of implementations.

**Summary**	**TDR**	**SIM**	**ASM**	**LDM**
Assay conditions minimizing the change in density due to growth	X			
Minimize incubation time necessary for producing transconjugants			X	
An incubation time results in a subset of parallel populations having no transconjugants				X
Assay occurs over a period of exponential cell growth		X*	X	X
Assay requires multiple parallel mating cultures to obtain one estimate				X
Assay requires a measurement of transconjugant density	X	X	X	
Assay requires a measurement of population growth rate		X*		
Assay requires a measurement of transconjugant growth rate			X	
* For the SIM assay, either the entire assay is conducted over exponentially growing cultures or an independent estimate for (maximum) population growth rate is needed.				

24. SI Fig. 5 – clarify which model has been simulated (equations 4.1-4.4?). Also add tD = tT = 0 (I assume) to the list of baseline parameter values.

We agree with the reviewer's suggested change. We have made the following highlighted word changes to SI Figure 5 legend (lines 448 – 460):

"**SI Figure 5 : The effect of incubation time ($\tilde{t}$) on estimating conjugation rate.** The Gillespie algorithm with equations [4.1]-[4.4] was used to simulate population dynamics. Donor conjugation rate for each parameter combination was estimated at 30-minute intervals (summarized using boxplots with the same graphical convention as in Figure 3). The gray dashed line indicates the true value for the donor conjugation rate (here, 10^{-14}). The baseline parameter values were $\psi_D = \psi_R = \psi_T = \psi_F = 1$, $\gamma_{DR} = \gamma_{DF} = \gamma_{TR} = \gamma_{TF} = 1 \times 10^{-14}$, and $\tau_D = \tau_T = 0$. The dynamic variables were initialized with $D_0 = R_0 = 10^5$ and $T_0 = F_0 = 0$. The LDM, SIM, TDR, and ASM estimates are in separate plots with estimate specific colors (brown, orange, cyan, and green, respectively). (a) Baseline parameters were simulated as the non-heterogenous parameter comparison. (b) An unequal growth rate was simulated with $\psi_D = \psi_T = 0.5$. (c) An unequal growth rate was simulated with $\psi_R = \psi_T = 2$. (d) An unequal conjugation rate was simulated with $\gamma_{TR} = 10^{-8}$. (e) A non-zero plasmid loss rate was simulated with $\tau_D = \tau_T = 0.0001$."

25. SI sections 4f-4h: I'd find it helpful to introduce these sections by briefly explaining the motivation/aim(s) of the following analysis. Similarly, at the end of section 4h, it would be helpful to add a conclusion – is there some additional insight into interpretation of experimental data?

We thank the reviewer for the useful suggestion. We agree that this would increase the readability. We have added the following highlighted words to the indicated sections:

SI lines 464 – 466 for SI section 4f:

"To investigate the incongruency observed between the SIM and LDM estimates in Figure 6, we extend equations [4.1]-[4.4] to incorporate batch culture dynamics by tracking the change in resource concentration."

SI lines 478 – 501 for SI section 4g:

"Here we used equations [4.5]-[4.12] which incorporates batch culture dynamics to simulate the cross-species case study with the experimentally measured parameters to investigate the incongruency observed between the SIM and LDM estimates in Figure 6. Most of the parameters were from the average of six experiments (D_0 = 1.17 x 10^5, R_0 = 3.33 x 10^4, ψ_D = 1.91, ψ_R = 1.47, ψ_T = 1.48, γ_{DR} = 1.96 x 10^{-13}, and γ_{TR} = 1.96 x 10^{-7}) with the remaining parameters informed by the 24 hour densities as to mimic the batch culture conditions of the experiment (C_0 = 4.41 x 10^9, Q = 1 x 10^7, and e = 1). We used the numerical solution to calculate the SIM estimate over time.

We compared the numerical solution to the actual experimental measurements from the cross-species experiments. The simulated density and conjugation estimate (SI Figure 6a solid lines) were similar to the average experimental densities and the experimental SIM estimate (SI Figure 6a circle data points). Thus, the experimental LDM estimates for the cross-species conjugation rates (γ_{DR} = 1.96 x 10^{-13}) and the within-species (γ_{TR} = 1.96 x 10^{-7}) along with the measured growth rates are sufficient to recapture a relatively inflated experimental SIM estimate. In contrast, a simulation with homogenous conjugation rates using either the cross- or within-species conjugation rate does not closely align with the experimental data (SI Figure 6b and c, respectively). These simulations also demonstrate that the heterogeneity in the measured growth rates is insufficient to produce the mismatch observed in the experimental data (SI Figure 6b and c). This was worth checking given that heterogeneity in growth rates violates a modeling assumption of the SIM approach. This adds further support that the parametric heterogeneity in the conjugation rates is the potential cause for the incongruency between the LDM and SIM estimates reported in Figure 6."

SI lines 518 – 538 for SI section 4h:

"In this section, we explored a violation of a modeling assumption in the SIM approach by using a model variation where the functional form of growth rate and conjugation rate are not proportional. This is relevant given that there are plasmid systems that will readily violate this proportional assumption (e.g., IncP plasmids). Here we assume that while growth rates follow the Monod equation, conjugation rates are not dependent on resource and remain constant after resources are depleted. We found that using this model and the same experimentally measured parameter values used in SI Figure 6 resulted in a higher SIM estimate at 24 hours (SI Figure 7a) compared to the scenario where conjugation rate is proportional to growth rate (SI Figure 7b). It is worth noting that by using this new model and these particular parameter values, the recipient pool is completely depleted which coincides with the SIM estimate no longer being a finite, positive value. This differs from SI Figure 7b where the SIM estimate hits an asymptote remaining at a finite, positive value. In this case, the recipient pool is not depleted because in this version of the model (SI section 4f) the conjugation rates approach zero as the resources are depleted. We acknowledge that a violation of the proportional assumption would lead to an inflation of the SIM estimate at 24 hours, which is the same pattern we show in our experimental results in Figure 6. However, we used an IncF plasmid in our experiment which was the plasmid system used in the original SIM study where the experimental results were consistent with a proportional relationship. We note that this analysis is relevant to other plasmid systems where this assumption is known to be violated or has not been experimentally validated."

26. SI Fig. 6 caption (L512, 514): "significantly" has connotations of having done a statistical test – rather say "substantially"?

We thank the reviewer for catching this mistake. We have removed and replaced the word "significantly" in the legend for Fig S6 with the highlighted word changes, which now reads (SI lines 511 – 514):

"(b) A scenario with homogenous low conjugation rates (γ_{DR} = γ_{TR} = 1.96 x 10^{-13}) deviates markedly from the experimental measurements. (c) A scenario with homogenous high conjugation rates (γ_{DR} = γ_{TR} = 1.96 x 10^{-7}) deviates substantially from the experimental measurements."

27. SI section 6b – around L636/SI Fig. 8b: the growth rate varies over time – which value was taken as the final estimate? The maximum?

We thank the reviewer for pointing this out. The purpose of the growth rate assays is to determine the incubation time necessary to get the donor and recipient strains in exponential growth before they are mixed at the start of the conjugation assay. We have made the following highlighted word changes to the relevant section to clarify (SI lines 646 – 645):

"The growth rates were calculated by taking the slope of each neighboring time point using equation [1.12] (SI Figure 8b). Using the growth rates calculated over time, an incubation time was chosen that coincided with the population growing at or near the maximum growth rate for each strain to ensure bacterial cultures entered the phase of maximal or close to maximal growth before the start of the conjugation assay. Thus, the growth rate estimates over time were used solely for determining the pre-assay growth period before the conjugation assay is executed and not to calculate the LDM estimate itself. A pre-assay growth period of 4 hours was used for both donors, E(pF) and K(pF), and the recipient, E(Ø)."

28. SI section 6c – please clarify, does the ratio of [Strep]/[Tet] remain constant in dual-antibiotic medium? (I.e., it's not a two-way gradient?)

We apologize for the lack of clarity. We have made the following highlighted word changes to the relevant section (SI lines 671 – 676):

"Then 500 µl of dual-antibiotic medium (streptomycin and tetracycline) was added to each well at increasing concentrations, forming a 2-fold gradient across the column. We note that the ratio of the two antibiotics was kept constant over the gradient. For each strain, this was repeated in three columns. After an overnight incubation, the well with the lowest concentration of the dual antibiotic medium across all replicates with no turbid growth was identified as the strain-specific MIC (SI Table 7)."

29. SI section 6d, L723: just to check, was the final density = 4 x 10^-7 x initial density (i.e. dilution factor 0.25 x 10^7) or was the dilution factor 4 x 10^7?

We thank the reviewer for pointing this out. We have made the following highlighted word changes to help clarify that it was the dilution factor (SI lines 731 – 734):

"In the laboratory, we used the protocol implemented by Alexander and MacLean to estimate π_x with a few adjustments. Briefly, the transconjugants were diluted (using a dilution factor of 4 x 10^{-7}) and 50 µl were dispersed into all wells in a deep-well microtiter plate."

30. SI section 6d, around L761: I'm confused about why this issue arises – why is the recipient colony count so low on plates supplemented with antibiotic selecting for the recipient?

Thanks for asking about this. The issue arises mainly due to the recipient and transconjugant densities becoming very similar and that the recipient-selecting agar plates permit both transconjugant and recipient growth. To explain in more detail, the colony counts of the recipient and transconjugant plates (which are always within normal range, ~20 to 300 colonies) are turned into densities using the colony counts and the dilution spread on the plate. We then change the estimated densities with the appropriate factor to correct for the extinction probabilities. Given that the transconjugant extinction probability on the transconjugant-selecting agar plates is higher than the recipient extinction probability on the recipient-selecting agar plates (i.e., 0.99 vs. 0.55, respectively), the transconjugant density increases relatively more than the recipient density. After the correction, we subtract the transconjugant density from the recipient density which results sometimes in a negative number given that transconjugants can grow on the recipient-selective plates (given that these plates are selecting for the host type). In other words, this is indicative that many or all the colonies growing on the recipient-selective plates may be transconjugants. To clarify why a negative value may arise, we added the following highlighted word to the relevant section (SI lines 764 – 780):

"Given the high transconjugant extinction probability on the transconjugant-selecting agar plates (see SI Table 9), the transconjugant density increases after the correction. Indeed, the transconjugant population can become more common than the "estimated" recipient population. We say "estimated" because there are no agar plates that select *only* for recipient cells. Specifically, the "recipient-selecting" agar plates allow for both recipient and transconjugant growth. To determine the recipient density, we subtract the transconjugant density from the density of cells calculated from the "recipient-selecting" agar plate counts. When the transconjugants are more abundant than—or at relatively similar densities to—recipients, the exact recipient density cannot be determined due to its relative scarcity. Specifically, the subtractive plating scheme could result in a negative value. We note that this happens rarely given that transconjugant densities are typically orders of magnitude lower than recipients. In the cases of high conjugation rates and long incubation times, this issue is more likely to arise. If the recipient density went negative after subtraction, then the non-subtracted recipient density was used instead. An overestimate for recipient density leads to an underestimate for the SIM estimate at 24 hours; therefore, the differences between the cross-species LDM and SIM estimates shown in Figure 6 are conservative."

31. L807: "10^4, 10^5, and 10^6-fold *dilution*" (missing word)

Thank you for catching the wrong word. We have added the following highlighted word to the relevant sentence (SI lines 819 – 820):

"Four columns were used for each initial density (10^4, 10^5, and 10^6 cells per ml) where 2 rows were used for each incubation time (0, 1, 2, and 3 hours) resulting in 8 wells per density-time treatment."

32. SI section 7 – Again, I'd find it helpful to start off with a statement of motivation/aim for this section.

We thank the reviewer for the useful suggestion. We agree that we could increase the readability. We have added the following highlighted words to the top of SI section 7 (SI lines 902 – 912):

"SI section 7: Probability generating function, low-order moments, and failure to establish

The aim of the first part of this section is to explore the connection between mutation and conjugation processes further. In the second part of this section, we derive a general expression for the LDM estimate that incorporates cases when the transconjugant doesn't always establish a successful lineage (i.e., non-zero extinction probability).

Keller and Antal (7) studied a generalization of the process explored by Luria and Delbrück (4). To start, Keller and Antal consider a wildtype population expanding from a single cell as follows:"

References:

1. J. S. Huisman, *et al.*, Estimating plasmid conjugation rates: a new computational tool and a critical comparison of methods. *bioRxiv*, 2020.03.09.980862 (2021).

2. A. E. Dewar, *et al.*, Plasmids do not consistently stabilize cooperation across bacteria but may promote broad pathogen host-range. *Nat Ecol Evol* **5**, 1624–1636 (2021).

3. S. Bates, A. M. Cashmore, B. M. Wilkins, IncP plasmids are unusually effective in mediating conjugation of Escherichia coli and Saccharomyces cerevisiae: involvement of the tra2 mating system. *J. Bacteriol.* **180**, 6538–6543 (1998).

4. S. E. Luria, M. Delbrück, Mutations of Bacteria from Virus Sensitivity to Virus Resistance. *Genetics* **28**, 491–511 (1943).

5. B. R. Levin, F. M. Stewart, V. A. Rice, The kinetics of conjugative plasmid transmission: fit of a simple mass action model. *Plasmid* **2**, 247–260 (1979).

6. H. K. Alexander, R. C. MacLean, Stochastic bacterial population dynamics restrict the establishment of antibiotic resistance from single cells. *Proc. Natl. Acad. Sci. U. S. A.* **117**, 19455–19464 (2020).

7. P. Keller, T. Antal, Mutant number distribution in an exponentially growing population. *J. Stat. Mech.* **2015**, P01011 (2015).
