# Supplementary material for: Estimating the transfer rates of bacterial plasmids with an adapted Luria–Delbrück fluctuation analysis
Source: PLoS Biol. 2022 Jul 25;20(7):e3001732. doi: 10.1371/journal.pbio.3001732 (PMC9352209; doi:10.1371/journal.pbio.3001732)
Supplement: Attachment — Submitted filename: Reviewer_Response_Letter.pdf [file pbio.3001732.s004.pdf]

June 26, 2022

Response to reviewers and editor

Data and other policy-related requests from the editor:

b) Thanks for providing the underlying data and code in GitHub. Please could you also deposit a permanent, unchangeable version in a repository like Zenodo that provides a DOI?

We have archived the GitHub repository on Zenodo (https://doi.org/10.5281/zenodo.6677158) and added this link in the main text in the appropriate places.

c) Please cite these (Github/Zenodo etc) clearly in each relevant main and supplementary Figure legend as the location of the data (e.g. "The data and code needed to generate this Figure can be found at https://github.com/livkosterlitz/LDM etc.").

We have added the Github/Zenodo links to the relevant figure legends in the main text and the supplement.

Reviewer 2:

I am satisfied with the author responses to my previous review and am very happy to recommend this manuscript for publication.

We thank the reviewer for this positive assessment.

Reviewer 3:

The authors have thoroughly addressed my previous comments. I especially like the additional analysis added to SI section 8 regarding optimising experimental design for a fixed total number of wells.

We thank the reviewer for their positive comments.

A few minor issues for final polishing:
- The authors could consider mentioning in the main text that the protocol is detailed on protocols.io

We are grateful to the reviewer for the suggestion. We have made the following changes (highlighted words) to the relevant section (lines 604 – 606):

“Materials and Methods
More detailed information for the mathematical models, simulations, and experiments are provided in the Supplementary Information. A general LDM protocol is deposited on protocols.io (dx.doi.org/10.17504/protocols.io.e6nvwk812vmk/v3).”

- Regarding my previous comment #13, the authors gave a detailed response with additional analysis, but unless I've overlooked something, it doesn't look like anything was changed in the manuscript. The authors could consider at least adding their final conclusion, that variances of the SIM and ASM estimates are approximately equal. (Perhaps the full derivation could be added to the GitHub Appendix?)

We agree that it would be a good idea to include the result from this additional analysis. We have made the following changes (highlighted words) to the relevant section (SI lines 1111 - 1114) and added the detailed response for this result in the relevant GitHub Appendix VII.

"In GitHub Appendix VII, we demonstrate this precision advantage for the LDM estimate mathematically. Also in GitHub Appendix VII, we derive an approximation for the variance for the SIM estimate, which demonstrates that the variances for the SIM and ASM estimates are extremely similar."

- In the newly added SI Figure 11:
In caption (a), variation --> variance?; "among the 100 estimates" rather than "of each estimate"?

We thank the reviewer for pointing these out. We made the following highlighted word changes to the relevant sentences in the legend for SI Figure 11 (SI lines 1195 - 1197):

"(a) The variance among the 100 estimates is given at 15-minute intervals where more than 1 out of the 100 calculated estimates produced a finite non-zero value. We ignore infinite estimates in the calculation of the variance."

In panel (d), how is \tilde{t} set?

We should have made this clearer. We made the following highlighted word changes to the relevant sentences in the legend for SI Figure 11 (SI lines 1202 - 1205):

"Each plotted point is the mean conjugation rate of the rates calculated for each group (where the number of populations within each group vary as indicated by the W value) at a specific incubation time ($\tilde{t} = 2.35$) selected using the criteria described in the Materials and Methods."

"Each partitioning was run 10 times" - was a new set of 500 populations simulated for each partitioning? (How else would you get variation among the 10 partitioning when W=500?)

We recognize that this part of the legend could have been made clearer. We made the following highlighted word changes to the relevant sentences in the legend for SI Figure 11 (SI lines 1205 – 1206):

"We ran the partitioning analysis 10 times using a new set of 500 populations"

- Regarding my previous comment #29: I'm guessing the authors mean to say a dilution factor of 4 x 10^7, not 10^-7?

We thank the reviewer for pointing this out. We made the following highlighted word change to the relevant sentence (SI lines 767 - 768):

"Briefly, the transconjugants were diluted (4×10^7 fold) and 50 μl aliquots were dispensed into all wells in a deep-well microtiter plate."
